# Characterisation of the blood RNA host response underpinning severity in COVID-19 patients

**DOI:** 10.1038/s41598-022-15547-2

**Published:** 2022-07-17

**Authors:** Heather Jackson, Irene Rivero Calle, Claire Broderick, Dominic Habgood-Coote, Giselle D’Souza, Samuel Nichols, Ortensia Vito, Jose Gómez-Rial, Carmen Rivero-Velasco, Nuria Rodríguez-Núñez, Gema Barbeito-Castiñeiras, Hugo Pérez-Freixo, Manuel Barreiro-de Acosta, Aubrey J. Cunnington, Jethro A. Herberg, Victoria J. Wright, Alberto Gómez-Carballa, Antonio Salas, Michael Levin, Federico Martinon-Torres, Myrsini Kaforou, PERFORM consortium, Heather Jackson, Irene Rivero Calle, Dominic Habgood-Coote, Giselle D’Souza, Samuel Nichols, Jose Gómez-Rial, Aubrey J. Cunnington, Jethro A. Herberg, Victoria J. Wright, Alberto Gómez-Carballa, Antonio Salas, Michael Levin, Federico Martinon-Torres, Myrsini Kaforou, GEN-COVID (www.gencovid.eu) study group, Aguilera Guirao Antonio, Álvarez Escudero Julián, Antela López Antonio, Barbeito Castiñeiras Gema, Bello Paderne Xabier, Ben García Miriam, Carral García María Victoria, Cebey López Miriam, Coira Nieto Amparo, Conde Pájaro Mónica, Costa Alcalde José Javier, Currás Tuala María José, Dacosta Urbieta Ana Isabel, Díaz Esteban Blanca, Domínguez Santalla María Jesús, Fernández Pérez Cristina, Fernández Villaverde Juan, Galbán Rodríguez Cristóbal, García Allut José Luis, García Vicente Luisa, Giráldez Vázquez Elena, Gómez Carballa Alberto, Gómez Rial José, González Barcala Francisco Javier, Guerra Liñares Beatriz, Leboráns Iglesias Pilar, Lence Massa Beatriz, Lendoiro Fuentes Marta, López Franco Montserrat, López Lago Ana, Martinón-Torres Federico, Navarro De la Cruz Daniel, Núñez Masid Eloína, Ortolá Devesa Juan Bautista, Pardo Seco Jacobo, Pazo Núñez María, Pérez del Molino Bernal Marisa, Pérez Freixo Hugo, Piñeiro Rodríguez Lidia, Pischedda Sara, Portela Romero Manuel, Pose Reino Antonio, Prada Hervella Gloria María, Queiro Verdes Teresa, Redondo Collazo Lorenzo, Regueiro Casuso Patricia, Rey García Susana, Rey Vázquez Sara, Riveiro Blanco Vanessa, Rivero Calle Irene, Rivero Velasco Carmen, Rodríguez Núñez Nuria, Rodríguez-Tenreiro Sánchez Carmen, Saborido Paz Eva, Sadiki Orayyou José Miguel, Saito Villanueva Carla, Serén Fernández Sonia, Souto Sanmartín Pablo, Taboada Muñiz Manuel, Trastoy Pena Rocío, Treviño Castellano Mercedes, Valdés Cuadrado Luis, Varela García Pablo, Vilas Iglesias María Soledad, Viz Lasheras Sandra, Ferreiro-Iglesias Rocio, Bastón-Rey Iria, Calviño-Suárez Cristina

**Affiliations:** 1 Section of Paediatric Infectious Disease, Faculty of Medicine, Imperial College London, London, SW7 2AZ UK; 2 Centre for Paediatrics and Child Health, Imperial College London, London, SW7 2AZ UK; 3 Translational Pediatrics and Infectious Diseases, Pediatrics Department, Hospital Clínico Universitario de Santiago, Santiago de Compostela, Spain; 4 Genetics, Vaccines, Infectious Diseases and Pediatrics Research Group (GENVIP;), Instituto de Investigación Sanitaria de Santiago, Universidad de Santiago de Compostela, Galicia, Spain; 5 Laboratorio de Inmunologìa, Servicio de Análisis Clìnicos, Hospital Clìnico Universitario Santiago de Compostela, Servizo Galego de Saúde, Galicia, Spain; 6 Intensive Medicine Department, Hospital Clìnico Universitario de Santiago de Compostela, Galicia, Spain; 7 Pneumology Department, Hospital Clìnico Universitario de Santiago de Compostela, Galicia, Spain; 8 Microbiology Department, Hospital Clìnico Universitario de Santiago de Compostela, Galicia, Spain; 9 Preventive Medicine Department, Hospital Clínico Universitario de Santiago de Compostela, Galicia, Spain; 10 Digestive Department, Hospital Clìnico Universitario de Santiago de Compostela, Galicia, Spain; 11 Unidade de Xenética, Instituto de Ciencias Forenses, Facultade de Medicina, Universidade de Santiago de Compostela, and GenPoB Research Group, Instituto de Investigación Sanitaria (IDIS), Hospital Clìnico Universitario de Santiago, Servizo Galego de Saúde, Galicia, Spain; 12 Anesthesiology and Resuscitation Service, Hospital Clínico Universitario de Santiago, Santiago de Compostela, Galicia, Spain; 13 Internal Medicine Service, Hospital Clínico Universitario de Santiago, Santiago de Compostela, Galicia, Spain; 14 Director of Nursing Processes, Hospital Clínico Universitario de Santiago, Santiago de Compostela, Galicia, Spain; 15 Manager of the Health Care Area of Santiago de Compostela and Barbanza, Hospital Clínico Universitario de Santiago, Santiago de Compostela, Galicia, Spain; 16 Clinical Chemistry Laboratory, Hospital Clínico Universitario de Santiago, Santiago de Compostela, Galicia, Spain

**Keywords:** Bioinformatics, Computational biology and bioinformatics, Systems biology, Diseases

## Abstract

Infection with SARS-CoV-2 has highly variable clinical manifestations, ranging from asymptomatic infection through to life-threatening disease. Host whole blood transcriptomics can offer unique insights into the biological processes underpinning infection and disease, as well as severity. We performed whole blood RNA Sequencing of individuals with varying degrees of COVID-19 severity. We used differential expression analysis and pathway enrichment analysis to explore how the blood transcriptome differs between individuals with mild, moderate, and severe COVID-19, performing pairwise comparisons between groups. Increasing COVID-19 severity was characterised by an abundance of inflammatory immune response genes and pathways, including many related to neutrophils and macrophages, in addition to an upregulation of immunoglobulin genes. In this study, for the first time, we show how immunomodulatory treatments commonly administered to COVID-19 patients greatly alter the transcriptome. Our insights into COVID-19 severity reveal the role of immune dysregulation in the progression to severe disease and highlight the need for further research exploring the interplay between SARS-CoV-2 and the inflammatory immune response.

## Introduction

Since severe acute respiratory syndrome coronavirus 2 (SARS-CoV-2), the causative agent of coronavirus disease 19 (COVID-19), emerged in Wuhan (China) in late 2019, it has caused considerable morbidity and mortality, in addition to major impacts on global health and economic systems. Manifestation of SARS-CoV-2 infection is highly heterogeneous, with some individuals remaining asymptomatic and others developing COVID-19, which can range from mild flu-like symptoms to severe life-threatening disease requiring mechanical ventilation and intensive care, and even death^1^. Most SARS-CoV-2 infections lead to mild symptoms or no symptoms at all, allowing cases to remain undetected and thus facilitating its spread throughout populations^1,2^. It was recognised early during the pandemic that age and pre-existing health conditions, such as obesity and diabetes, are amongst the main risk factors for developing severe COVID-19^3,4^. Dysfunctional immune responses to SARS-CoV-2, specifically impaired type I interferon responses in conjunction with an exacerbated inflammatory response, have been associated with progression to severe COVID-19^5–7^. An imbalanced immune response leads to the development of a ‘cytokine storm’ which causes lung inflammation, septic shock, and multi-organ failure^5,7,8^.

Identification of improved treatments and prevention of severe COVID-19 requires better understanding of the underlying immune and inflammatory processes that distinguish severe disease from mild illness. Host whole blood transcriptomic profiling of patients with infectious and inflammatory conditions has been extensively used for understanding infectious disease dynamics, from the identification of accurate biomarkers of infection^9–11^ to gaining insights into variations in the host response to different pathogens and severity of disease^12,13^. Targeted^6,14^ and untargeted^15–18^ transcriptomic profiling of whole blood from SARS-CoV-2-positive hosts has already been undertaken. To the best of our knowledge, severity of COVID-19 has not yet been explored in whole blood with non-hospitalised SARS-CoV-2-positive cases included as a comparator group, nor has it been explored across a range of severities. Furthermore, the impacts of severity on the transcriptome have not yet been explored in an approach that accounts for the treatment regiments received by the patients. The study of transcriptomic profiles from individuals with varying severities will be an essential tool for improving our understanding of the course of disease resulting from SARS-CoV-2 infection.

We have analysed the whole blood transcriptomes obtained from individuals with different levels of COVID-19 severity to explore how the host blood transcriptome changes with increasing COVID-19 severity, aiming to identify the key biological processes and genes underpinning differences in severity.

## Methods

### Study design and clinical cohort

Adult patients with COVID-19 were recruited through the GEN-COVID study group (www.gencovid.eu) at Hospital Clínico Universitario de Santiago de Compostela (Galicia, Spain) between March 2020 and May 2020. COVID-19 was defined according to the current national guidelines in Spain (https://www.mscbs.gob.es/profesionales/saludPublica/ccayes/alertasActual/nCov/documentos.htm).

Subjects granted informed consent for their participation in the study. If this was not possible at the time of sampling, deferred consent was allowed, and subjects were approached for consent at the earliest appropriate opportunity. Subjects who did not agree to participate in the study were excluded. GEN-COVID Study was approved by the Ethics Committee of Galicia by fast-track procedure on 18^th^ March 2020 (CEIC Galicia, reg 2020/178).

Patients with COVID-19 were categorised as having mild, moderate, or severe disease. Mild patients were those who were always outpatients; emergency department attendance was the ceiling of care, and they were not admitted to hospital (WHO score 1–2). Moderate patients were those admitted to hospital, for whom ward-based therapy was the ceiling of care with supportive care limited to oxygen delivery; no intensive care unit (ICU) admission (WHO score 3–4). Severe patients were those who were admitted to ICU at any time throughout the course of their disease (WHO score 5–7). Supportive care included high flow oxygen (> 16 L/ minute), non-invasive ventilation (NIV), invasive ventilation, inotropic support, renal replacement therapy, and extracorporeal membrane oxygenation (ECMO). The severe category also included those patients who died (WHO score 8), in the emergency department, ward or ICU. Research blood samples were taken following admission to hospital for moderate and severe COVID-19 patients. For mild patients, patients were recruited via telephone following a positive SARS-CoV-2 test, and visited at home by the research team to obtain a research blood sample and consent.

### RNA isolation and quantification

Whole blood was collected at the time of recruitment into PAXgene blood RNA tubes (PreAnalytiX), frozen, and total RNA (including RNA > 18 nucleotides) was isolated according to the manufacturer’s instructions (Qiagen). RNA samples were stored at − 80 °C, before undergoing an additional DNAse treatment using an RNA clean & concentrator kit (Zymo Research) prior to sequencing at The Wellcome Centre for Human Genetics in Oxford, UK. Material was quantified using RiboGreen (Invitrogen) on the FLUOstar OPTIMA plate reader (BMG Labtech) and the size profile and integrity analysed on the 2200 TapeStation (Agilent, RNA ScreenTape). Input material was normalised and strand specific library preparation was completed using NEBNext® Ultra™ II mRNA kit (NEB) and NEB rRNA/globin depletion probes following manufacturer’s instructions. Libraries were on a Tetrad (Bio-Rad) using in-house unique dual indexing primers (based on^19^). Individual libraries were normalised using Qubit and pooled together. The pooled library was diluted to ~ 10 nM for storage and denatured and further diluted prior to loading on the sequencer. Paired end sequencing was performed using a Novaseq6000 platform at 150 paired end configuration. The RNA-Seq analysis pipeline consisted of quality control using FastQC^20^, MultiQC^21^ and annotations modified with BEDTools^22^, alignment and read counting using STAR^23^, SAMtools^24^, FeatureCounts^25^ and version 89 ensembl GCh38 genome and annotation^26^.

### Statistical analysis

All statistical analyses were performed using the statistical software R (R version 4.0.3)^27^. Normalised counts were calculated for each gene using DESeq2 (V1.30.0)^28^ and default parameters were used. Normalised genes with fewer than three samples with a normalised read count of at least 20 were considered lowly expressed and were removed, leaving a total of 20,536 genes. Principal component analysis (PCA) was performed on the normalised counts.

Immune cell level measurements were not available for all individuals included in the analysis. Therefore, cell-type fractions were estimated from the bulk host transcriptome data using the CIBERSORTx algorithm^29^. The estimated fractions were compared across the three COVID-19 patient groups (mild, moderate, and severe) and statistical significance was evaluated using the Kruskal–Wallis test followed by the pairwise Dunn’s test with *p*-values adjusted using the Benjamini–Hochberg (BH) correction (moderate *vs.* mild; severe *vs.* mild; severe *vs.* moderate). Adjusted *p*-values < 0.05 were considered significant. Immune cell proportions were explored in relation to age and sex. Pairwise Mann–Whitney U tests were performed contrasting males *vs*. females within each severity group and generalised linear models (GLMs) were performed testing the relationship between age and immune cell proportions with each severity group, with *p*-values adjusted using the BH adjustment.

DESeq2^28^ was used for differential expression analysis (further information in Supplementary Methods). Prior to exploring the impact of COVID-19 severity on the transcriptome, the effect of immunomodulatory treatment on the transcriptome was assessed through differential expression analysis using DESeq2^28^. Patients with moderate COVID-19 who were not receiving steroids at the time of sampling were contrasted against patients with moderate COVID-19 who were receiving steroids at the time of sampling, whilst accounting for age and sex in the model. Steroids were chosen as the immunomodulatory treatment to explore due to adequate sample size in comparison to other immunomodulatory treatment groups such as tocilizumab. Equally, moderate COVID-19 samples were chosen due to the group size. Patients receiving tocilizumab or interferon therapies were excluded from this comparison. Treatments including antibiotics, antivirals and antimalarials were allowed.

To determine whether administration of antivirals influenced the host transcriptome, samples from moderate COVID-19 patients who received antivirals were contrasted against samples from moderate COVID-19 patients who did not receive antivirals using DESeq2^28^ with a model accounting for age and sex.

DESeq2^28^ was used for differential expression analysis of COVID-19 severity groups. We assessed transcriptomic differences with increasing COVID-19 severity by carrying out pairwise comparisons between each severity group (i.e., moderate *vs*. mild; severe *vs*. mild; and severe *vs*. moderate). We used two different model designs for each comparison. First, the models included immunomodulatory treatment status, sex, age, and severity. This model design aimed to account for transcriptomic differences induced by immunomodulatory treatments by including variables representing whether the patients received tocilizumab, steroids, or interferon treatment, in addition to sex, age and severity. The second model design included in silico immune cell proportion estimates, sex, age, and severity. This design accounted for transcriptomic differences induced by different proportions of immune cells, and it included the immune cell fractions described below as well as sex, age, and severity. The immune cell proportions accounted for included: monocytes, neutrophils, B cells (the sum of naïve and memory B cells and plasma cell proportions), CD4 T cells (the sum of the proportions of naïve CD4 T cells, resting and activated memory CD4 T cells, follicular helper T cells and regulatory T cells), CD8 T cells and natural killer (NK) cells (the sum of resting and activated NK cell proportions).

Adjusted p-values were calculated using the BH procedure^30^. The log_2_ fold-changes (LFC) and adjusted p-values of all genes were visualised using volcano plots. Concordance and discordance resulting from different model designs were visualised using cross plots. Genes with an adjusted p-value < 0.05 were considered significantly differentially expressed (SDE). The lists of SDE genes were subjected to pathway analysis using Ingenuity Pathway Analysis (IPA; QIAGEN Inc., https://www.qiagenbioinformatics.com/products/ingenuity-pathway-analysis). IPA was selected because it can predict directionality of pathways through knowledge of molecular functions, and it returns a *z*-score for each predicted pathway. Positive and negative *z*-scores indicate that the pathway is upregulated or downregulated, respectively, in the group of interest compared against the reference group. Z-scores are calculated using the log2 fold change values obtained by the differential expression analysis with higher absolute z-scores representing a larger degree of change (further details in Supplementary Methods).

Severity was also explored as an additive variable (mild = 0; moderate = 1; severe = 2), and by contrasting samples from hospitalised COVID-19 patients (moderate and severe) to samples from non-hospitalised COVID-19 patients (mild) using DESeq2, with full methods described in the Supplementary Methods.

## Results

**Figure 1:**
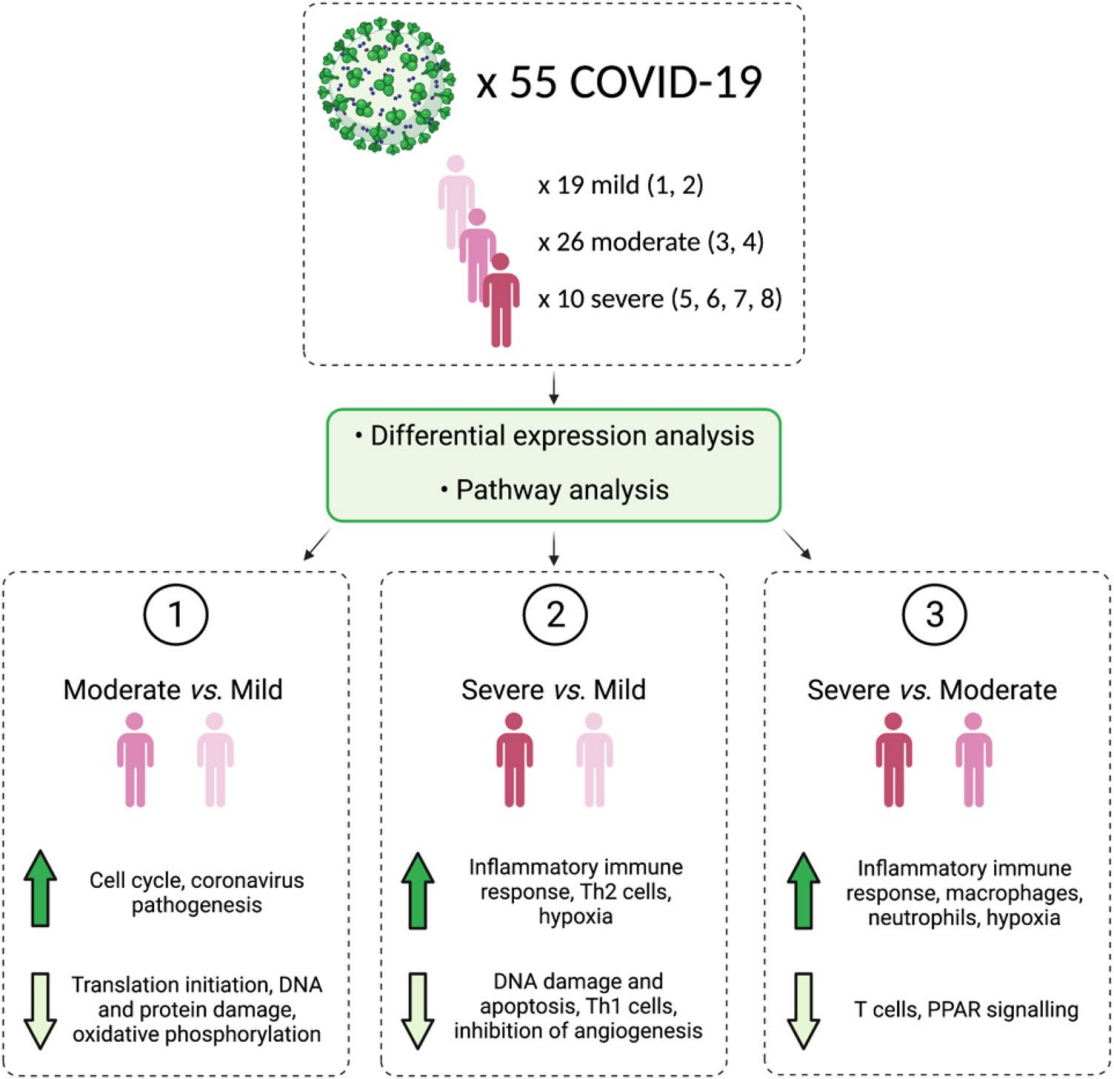
A schematic summarising the number of patients analysed, the main analysis steps, and the key findings. The numbers in brackets following mild, moderate, and severe are the WHO severity scores that make up these classifications. Figure made with BioRender (https://biorender.com/).

A schematic showing an overview of the patients, analysis, and key findings is shown in Fig. 1.

### Clinical description of patients

Whole blood transcriptomic profiling through RNA Sequencing (RNA-Seq) was performed on 65 samples from patients recruited through the GEN-COVID study. Samples from patients in whom a pathogen in addition to SARS-CoV-2 was isolated less than 5 days before or 10 days after the research blood sample were not selected (*n* = 10), as the aim was to identify gene expression changes in blood that reflect COVID-19 disease and not coinfections. Of the 55 remaining COVID-19 patients, 19, 26 and 10 patients had mild, moderate, and severe disease, respectively. In all but two cases, the severity categorisation of the patient matched their level of supportive care at the time the research blood sample. For these two patients, the decision to transfer them to ICU was made within 36 h of the sample extraction, therefore they were classified as severe in our analyses.

The research blood sample was taken at home for all mild COVID-19 patients since they were non-hospitalised. Following a positive SARS-CoV-2 test, mild patients were contacted via telephone and visited by the study team at home where the research blood sample was taken. For moderate and severe COVID-19 cases, the research blood sample was taken following admission to hospital at a median of 5 days (IQR: 4–7) and 7 days (IQR: 2.8–8.8) following admission for moderate and severe COVID-19, respectively (Table 1).

**Table 1:** Clinical characteristics of the patients included in the analysis (*n* = 55) stratified by COVID-19 severity.

	Mild (n = 19)	Moderate (n = 26)	Severe (n = 10)
**General demographics**			
Sex (female)	14 (73.7%)	12 (46.2%)	3 (30%)
Age (median)	37 (32.5–42.5)	69 (56.5–76.8)	65 (54.3–70)
< 50 years	19 (100%)	5 (19.2%)	0 (0%)
50–70 years	0 (0%)	9 (34.6%)	6 (60%)
> 70 years	0 (0%)	12 (46.2%)	4 (40%)
Ethnicity (South European)	19 (100%)	25 (96.2%)	10 (100%)
**Comorbidities**			
Endocrine	4 (21%)	14 (53.8%)	7 (70%)
Smoking	1 (5.3%)	4 (15.4%)	5 (50%)
Malignancy	0 (0%)	0 (0%)	1 (10%)
Pulmonary	0 (0%)	8 (30.8%)	1 (10%)
Gastrointestinal	0 (0%)	5 (19.2%)	1 (10%)
Neurological	3 (15.8%)	2 (7.7%)	0 (0%)
Cardiac	0 (0%)	7 (26.9%)	0 (0%)
Hypertension	1 (5.3%)	6 (23.1%)	3 (30%)
Immunosuppressed	0 (0%)	1 (3.8%)	1 (10%)
Hematology	0 (0%)	0 (0%)	0 (0%)
Allergic/autoimmune/inflammatory	2 (10.5%)	0 (0%)	0 (0%)
Genetic	0 (0%)	1 (3.8%)	0 (0%)
Organ transplant	0 (0%)	0 (0%)	1 (10%)
Obesity	1 (5.3%)	12 (46.2%)	5 (50%)
Pregnant	1 (5.3%)	0 (0%)	0 (0%)
Other	0 (0%)	0 (0%)	0 (0%)
**Admission timelines**			
Days since symptom onset (median, IQR)	11 (9.25–16.75)	15 (11–17)	10.5 (9–13.5)
Days between admission and sample extraction (median, IQR)	NA (all samples taken at home)	5 (4–7)	7 (2.8–8.8)
**Presenting symptoms**			
Fever	9 (47.4%)	22 (84.6%)	10 (100%)
Respiratory	12 (63.2%)	24 (92.3%)	9 (90%)
Cardiac	0 (0%)	1 (3.8%)	0 (0%)
Gastrointestinal	2 (10.5%)	9 (34.6%)	4 (40%)
Musculoskeletal	9 (47.4%)	16 (61.5%)	6 (60%)
Rash	0 (0%)	0 (0%)	0 (0%)
Sensory (ageusia, anosmia)	9 (47.4%)	8 (30.8%)	1 (10%)
Headache	10 (52.6%)	4 (15.4%)	0 (0%)
Shock	0 (0%)	0 (0%)	0 (0%)
Ill appearance	0 (0%)	2 (7.7%)	1 (10%)
**Investigations**			
White cells (10^9^/L)—mean (IQR)	Unknown	7 (4.6–6.9)	7.5 (5.2–10)
Neutrophils (10^9^/L)—mean (IQR)	Unknown	4.5 (2.6–4.7)	6.9 (4.5–9.1)
Lymphocytes (10^9^/L)—mean (IQR)	Unknown	1.6 (1.1–2.1)	0.65 (0.5–0.7)
Monocytes (10^9^/L)—mean (IQR)	Unknown	0.41 (0.32–0.51)	0.26 (0.14–0.38)
Platelets (10^9^/L)—mean (IQR)	Unknown	317.1 (242.5–381)	224.9 (128.8–340)
Fibrinogen (mg/dL)—mean (IQR)	Unknown	475.6 (476.5–500)	444.6 (379.5–511.5)
D-dimer (ng/mL)—mean (IQR)	Unknown	1694.7 (526–1132)	4122 (708–6267)
CRP (mg/L)—mean (IQR)	Unknown	34.4 (16.2–36.4)	81.8 (29.1–127.8)
Ferritin (μg/L)—mean (IQR)	Unknown	664.5 (255.8–734)	1604 (1196–1817)
**SARS-CoV-2 infection acquisition (if known)**			
Community acquired infection	18 (94.7%)	25 (96.2%)	10 (100%)
Hospital acquired infection	0 (0%)	0 (0%)	0 (0%)
**Treatment**			
Any antibiotics	0 (0%)	26 (100%)	10 (100%)
Macrolides (azithromycin)	0 (0%)	26 (100%)	10 (100%)
Cephalosporins (ceftriaxone, cefepime)	0 (0%)	15 (57.7%)	7 (70%)
Penicillin and beta-lactamase inhibitors (piperacillin tazobactam)	0 (0%)	1 (3.8%)	3 (30%)
Oxazolidinones (linezolid)	0 (0%)	0 (0%)	1 (10%)
Lincomycin (clindamyclin)	0 (0%)	0 (0%)	1 (10%)
Antiviral (lopinavir-ritonavir)	0 (0%)	22 (84.6%)	0 (0%)
Antimalarial (hydroxychloroquine)	0 (0%)	25 (96.2%)	10 (100%)
Steroids	0 (0%)	7 (26.9%)	5 (50%)
Monoclonal antibody (tocilizumab, infliximab, vedolizumab)	0 (0%)	1 (3.8%)	4 (40%)
**Supportive care required at time of sampling**			
Oxygen	0 (0%)	15 (57.7%)	0 (0%)
High-flow oxygen	0 (0%)	0 (0%)	5 (50%)
Non-invasive ventilation	0 (0%)	0 (0%)	1 (10%)
Invasive ventilation	0 (0%)	0 (0%)	4 (40%)
Inotropes	0 (0%)	0 (0%)	2 (20%)
ECMO	0 (0%)	0 (0%)	0 (0%)
Renal replacement	0 (0%)	0 (0%)	0 (0%)
Hemofiltration	0 (0%)	0 (0%)	0 (0%)
**Maximum level of care/outcome**			
Ambulatory	19 (100%)	0 (0%)	0 (0%)
Admitted to ward	0 (0%)	26 (100%)	0 (0%)
Admitted to ICU	0 (0%)	0 (0%)	10 (100%)
Died	0 (0%)	0 (0%)	0 (0%)

The section ‘Supportive care required information’ refers to the care required at sampling whilst ‘Maximum level of care/outcome information’ refers to the maximum level of care required for the patients after sampling. High-flow oxygen is oxygen therapy between 16 and 60 L/min.

Characteristics of the 55 COVID-19 patients are summarised in Table 1 and their WHO Severity Classification Score and how it relates to the severity groups used here is detailed in Table 2. 52.7% (*n* = 29) were female and 98% (*n* = 54) were South European. The median age was 55 years. 78.2% (*n* = 43) of the patients reported comorbidities with endocrine conditions (most frequently diabetes) as the most common comorbidities (45.4%; *n* = 25) followed by obesity (32.7%; *n* = 18) and hypertension (18.2%; *n* = 10). The rates of endocrine comorbidities, smoking and obesity were the highest in the severe group, with 70% (*n* = 7), 50% (*n* = 5), and 50% (*n* = 5) severe patients reporting endocrine conditions, smoking, and obesity, respectively, as comorbidities compared to 53.8% (*n* = 14), 15.4% (*n* = 4), and 46.2% (*n* = 12) of moderate patients, and 21% (*n* = 4), 5.3% (*n* = 1), and 5.3% (*n* = 1) for mild COVID-19. The duration of symptoms before presentation to Emergency Department (ED) was a median of 13 days. Main presenting symptoms were respiratory (80.3%; *n* = 45), fever (74.5%; *n* = 41) and musculoskeletal (56.4%; *n* = 31). All patients had PCR-confirmed SARS-CoV-2 infection and 96.4% (*n* = 53) were community acquired.

**Table 2:** WHO Severity Classification Score for the COVID-19 patients included in the analysis.

WHO Severity Classification	Mild COVID-19 (n = 19)	Moderate COVID-19 (n = 26)	Severe COVID-19 (n = 10)
Uninfected	0 (0%)	0 (0%)	0 (0%)
**Ambulatory**			
1—No limitation of activities	19 (100%)	0 (0%)	0 (0%)
2—Limitation of activities	0 (0%)	0 (0%)	0 (0%)
**Hospitalised, Mild Disease**			
3—Hospitalised, no oxygen therapy	0 (0%)	5 (19.2%)	0 (0%)
4—Oxygen by mask or nasal prongs	0 (0%)	21 (80.8%)	0 (0%)
**Hospitalised, Severe Disease**			
5—Non-invasive ventilation or high-flow oxygen	0 (0%)	0 (0%)	4 (40%)
6—Intubation and mechanical ventilation	0 (0%)	0 (0%)	3 (30%)
7—Ventilation + additional organ support- pressors, renal replacement therapy, ECMO	0 (0%)	0 (0%)	3 (30%)
8—Death	0 (0%)	0 (0%)	0 (0%)

This is the patients’ final severity score rather than their severity at the time of sampling (as shown in Table 1).

A combined triple therapy (antibiotic + antiviral [lopinavir-ritonavir] + antimalarial [hydroxychloroquine]) was the most common treatment administered to patients (*n* = 30; 54.5%). 21.8% (*n* = 12) received steroids and 9% (*n* = 5) received tocilizumab during their disease. 54.5% (*n* = 30) required oxygen at some point throughout the entire course of their disease, 7% (*n* = 4) required invasive ventilation and 3.6% (*n* = 2) required inotropes. Regarding outcomes, 34.5% (*n* = 19) were ambulatory patients, 47% (*n* = 26) were admitted to the ward, 18.2% (*n* = 10) were admitted to ICU, and none died. Supplementary Figure S1 shows that whilst the samples are clearly stratified by severity in PC1, there is confounding between severity and sex/age, a pattern which is also clear in Table 1.

### In silico immune cell proportion estimates

As COVID-19 is associated with changes in blood cell proportions, particularly between approximately days 4–14^31–33^, that are more prominent among critically ill patients with COVID-19^34,35^, we assessed the levels of the cell-type proportions estimated in silico from the RNA-Seq count data (Supplementary Fig. S2). The Kruskal–Wallis test was applied to determine whether the immune cell proportions for each severity group were derived from the same distribution. Significant p-values (*p*-value < 0.05) were reported for each immune cell type except neutrophils (*p*-value: 0.11) with the following *p*-values: CD4 T cells: 1.594 × 10^–5^; CD8 T cells: 1.738 × 10^–3^; B cells: 2.378 × 10^–3^; monocytes: 5.414 × 10^–4^; and NK cells: 1.468 × 10^–2^. The Dunn’s test was then applied to all cell types to determine whether any pairwise comparisons were significant (Supplementary Fig. S2). CD4 T cell proportions were significantly different between all three pairwise severity comparisons with levels decreasing with increasing severity (moderate *vs*. mild *p*-value = 4.62 × 10^–03^; severe *vs*. mild *p*-value = 1.52 × 10^–05^; severe *vs*. moderate *p*-value = 0.02). The proportions of CD8 T cells and NK cells were significantly different between severe *vs*. mild COVID-19 (CD8 T cell *p*-value = 1.11 × 10^–03^, NK cell *p*-value = 0.02) and severe *vs*. moderate COVID-19 (CD8 T cell *p*-value = 0.04, monocyte *p*-value = 1.59 × 10^–03^, NK cell *p*-value = 0.02). CD8 T cell, monocyte and NK cell proportions decreased between severe COVID-19 and mild and/or moderate COVID-19 whilst neutrophil proportions increased between mild /moderate and severe cases. Despite the neutrophils not reaching significance in the Kruskal–Wallis test, significant differences were observed between severe *vs*. mild (*p*-value = 7.10 × 10^–04^) and severe *vs*. moderate (*p*-value = 1.24^–03^), with levels increasing in severe COVID-19 compared to both moderate and mild. Since many mild patients were missing clinical cell counts, the in silico immune cell proportion estimates were used in downstream analyses in lieu of clinical cell counts.

### The effect of immunomodulatory treatment on COVID-19 patients’ blood transcriptome

Patients received various types of clinical interventions and treatments (Table 1), including immunomodulatory therapies. To assess the impact of immunomodulatory therapies on the transcriptome of individuals with COVID-19, we identified genes SDE between patients with moderate COVID-19 who did not receive steroids (*n* = 19) and patients with moderate COVID-19 who did receive steroids, excluding those who also received monoclonal antibody therapy (*n* = 6). Individuals who received steroids in combination with antibiotics, antivirals and antimalarials were included in the comparisons (Supplementary Table S2). 556 genes were SDE in patients who received steroids *vs*. those who did not (BH *p*-value < 0.05), of which 253 genes were over-expressed with steroid administration and 303 were under-expressed (Supplementary File 1). No significant pathways were identified by IPA.

84.6% (*n* = 22) of the moderate COVID-19 patients were administered antivirals (lopinavir-ritonavir). The transcriptome profiles of moderate COVID-19 patients who received antivirals were contrasted against the transcriptome profiles of moderate COVID-19 patients who did not receive antivirals (*n* = 24) to determine whether antiviral administration influenced the transcriptome. Using a DESeq2 model accounting for age and sex, only one SDE gene was identified as SDE between moderate COVID-19 patients who did and did not receive antivirals (lopinavir-ritonavir). The gene was DND1P1 (BH *p*-value: 0.040; LFC: 0.989). DND1P1 was not significant in any downstream analyses.

### Differential gene expression analysis of COVID-19 severity

**Figure 2:**
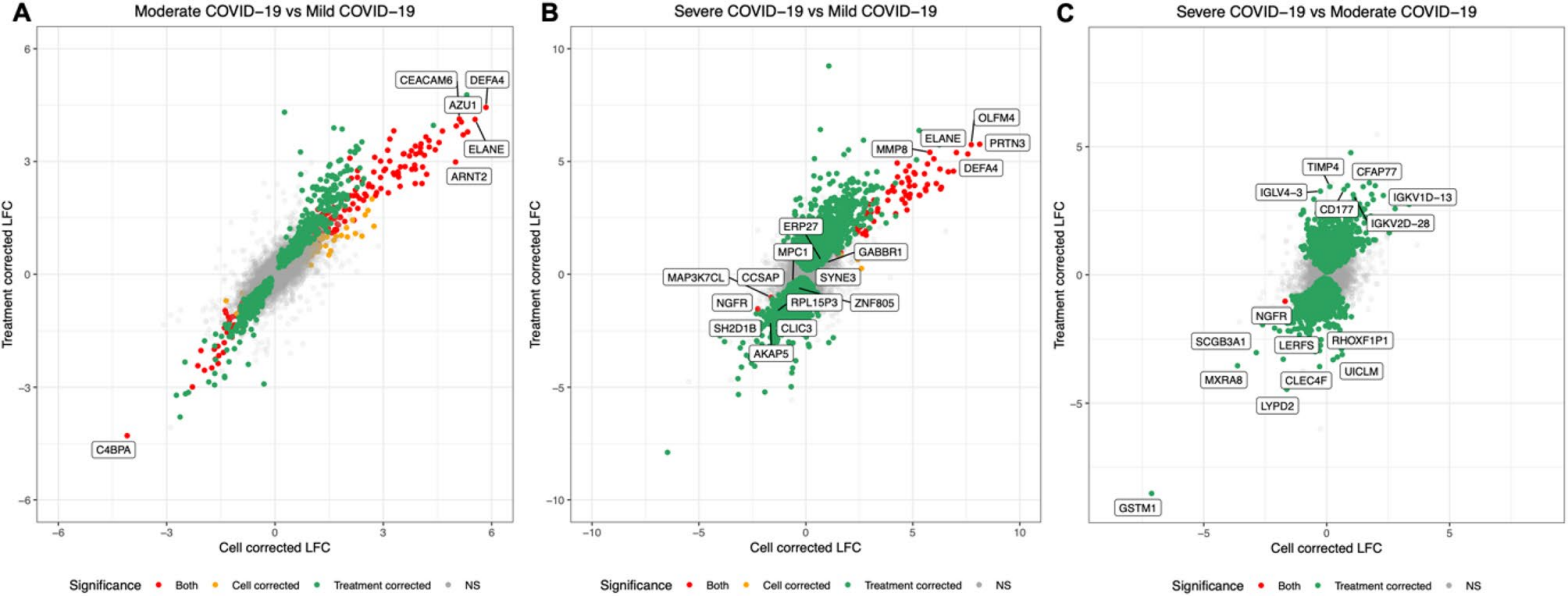
Cross plots showing the log_2_ fold change (LFC) values of genes for pairwise comparisons between three severity groups (**A**: moderate *vs.* mild; **B**: severe *vs.* mild; **C**: severe *vs.* moderate). The plots show how LFC values differ according to whether immune cell proportions (x-axis) or immunomodulatory treatments (y-axis) were included in the models. Red points are genes that were SDE in both models, whilst orange and green points are genes SDE in the cell correction and treatment correction models, respectively. *NS* not significant.

Pairwise comparisons were made between the three severity groups (mild, moderate and severe) to identify transcriptomic differences with increasing severity. In the moderate *vs*. mild COVID-19 comparison, there was greater concordance between the models accounting for immunomodulatory treatment and immune cell proportions as the number of genes identified as SDE in both models was higher than for the severe *vs*. mild and the severe *vs.* moderate comparisons (Fig. 2).

### Moderate COVID-19 vs. mild COVID-19

1547 genes were SDE between moderate (*n* = 26) and mild (*n* = 19) patients whilst accounting for immunomodulatory treatment, with 603 and 944 genes over- and under-expressed with increasing severity respectively (Supplementary File 2). When these genes were subjected to pathway analysis, EIF2 Signalling was the most significant pathway reduced in moderate cases compared to mild cases (*z*-score = − 5.778, BH *p*-value = 5.012 × 10^–29^) and Regulation of eIF4 and p70S6K Signalling (*z-*score = − 1, BH *p*-value = 1.000 × 10^–13^) were also found to be significantly enriched (Supplementary Table S3).

The Coronavirus Pathogenesis pathway was upregulated in moderate COVID-19 (*z-*score = 2.744, BH *p*-value = 5.248 × 10^–07^) in addition to various pathways related to the cell cycle including Mitotic Roles of Polo-Like Kinase (*z*-score = 0.816, BH *p*-value = 2.754 × 10^–05^), Cyclins and Cell Cycle Regulation (*z*-score = 2.714, BH *p*-value = 4.266 × 10^–02^) and Cell Cycle Control of Chromosomal Replication (*z*-score = 0.632; BH *p*-value = 2.042 × 10^–02^).

PAK signalling (*z*-score = − 2.53; BH *p*-value = 4.266 × 10^–02^) and mTOR signalling (*z*-score = − 0.632; BH *p*-value = 4.467 × 10^–08^) pathways were both found to be downregulated in moderate COVID-19. Two pathways related to aberrant protein production and DNA damage were identified as downregulated in moderate COVID-19 whilst accounting for treatment: Unfolded Protein Response (*z*-score = − 1; BH *p*-value = 1.514 × 10^–03^); and Role of CHK Proteins in Cell Cycle Checkpoint Control (*z*-score = − 2.828; BH *p*-value = 7.943 × 10^–03^). Oxidative phosphorylation was also identified by IPA as downregulated in moderate COVID-19 (*z*-score = − 4.243; BH *p*-value = 1.514 × 10^–03^).

488 genes were SDE between moderate and mild COVID-19 whilst accounting for immune cell proportions, with 222 and 266 genes over- and under-expressed respectively with increasing severity. 389 genes were SDE between moderate and mild COVID-19 irrespective of the model design (Fig. 2A). When immune cell proportions were included in the model, IPA identified two significant pathways (EIF2 Signalling: *z*-score = − 2.53, BH *p* value = 1.288 × 10^–03^; Regulation of eIF4 and p70S6K Signalling: BH *p*-value = 1.950 × 10^–02^; indeterminate z-score).

### Severe COVID-19 vs. mild COVID-19

7343 genes were SDE between severe (*n* = 10) and mild COVID-19 (*n* = 19) whilst accounting for immunomodulatory treatment with 3329 and 4014 genes over- and under-expressed with increasing severity, respectively (Supplementary File 3). 94 genes were SDE between severe and mild COVID-19 whilst accounting for immune cell proportions with 81 and 13 genes over- and under-expressed with increasing severity, respectively. 87 genes were SDE between severe and mild COVID-19 irrespective of the model design (Fig. 2B).

The pathways upregulated in severe COVID-19 were dominated by those related to the immune response, notably the inflammatory immune response (Supplementary Table S4). For example, TREM1 Signalling (*z*-score = 3.157; BH *p*-value = 3.162 × 10^–02^), STAT3 Pathway (*z*-score = 0.949; BH *p*-value = 7.762 × 10^–03^) and IL-22 signalling (*z*-score = 1.732; BH *p*-value = 2.239 × 10^–02^) were all amongst the pathways upregulated in severe COVID-19 compared to mild COVID-19. HIF1α Signalling was also identified by IPA following treatment correction (IPA *z*-score = 2.255; BH *p*-value = 3.890 × 10^–03^).

Inhibition of Angiogenesis by TSP1 was found to be downregulated by IPA whilst correcting for immunomodulatory treatment (IPA *z*-score = − 0.258; BH *p*-value = 7.762 × 10^–03^), whilst PDGF signalling (*z*-score = 1.414; BH *p*-value = 4.365 × 10^–02^) was found to be upregulated in severe COVID-19. Interestingly, the Th2 pathway (*z*-score = 1.372; BH *p*-value = 1.549 × 10^–04^) was upregulated in severe COVID-19 whilst the Th1 pathway (*z*-score = − 0.949; BH *p*-value = 7.413 × 10^–04^) was downregulated.

Following immunomodulatory treatment correction, there were several pathways downregulated in severe COVID-19 that were related to DNA damage and apoptosis, including NER (Nucleotide Excision Repair, Enhanced Pathway, *z*-score = − 2.744; BH *p*-value = 1.318 × 10^–02^), Role of BRCA1 in DNA Damage Response (*z*-score = − 0.943; BH *p*-value = 4.571 × 10^–02^) and TWEAK signalling (*z*-score = − 0.5; BH *p*-value = 3.715 × 10^–02^). Furthermore, the translation pathways EIF2 Signalling (IPA *z*-score = − 6.14; BH *p*-value = 1.259 × 10^–19^) and Regulation of eIF4 and p70S6K Signalling (IPA *z*-score = − 0.728; BH *p*-value = 1.230 × 10^–08^) identified in the moderate *vs.* mild comparison remained significantly downregulated.

One pathway was enriched following immune cell proportion correction: Airway Pathology in Chronic Obstructive Pulmonary Disease (B-H *p*-value = 4.571 × 10^–04^) with an indeterminate z-score.

### Severe COVID-19 vs. moderate COVID-19

8971 genes were SDE between severe and moderate COVID-19 whilst accounting for immunomodulatory treatment with 4380 and 4591 genes over- and under-expressed with increasing severity, respectively (Supplementary File 4). One gene (*NGFR*) was SDE between severe and moderate cases whilst accounting for immune cell proportions. *NGRF* was under-expressed in severe COVID-19 (BH *p*-value = 3.172 × 10^–02^; LFC = − 1.692).

Inflammatory pathways were observed as upregulated by IPA (Supplementary Table S5), such as Natural Killer Cell Signalling (*z*-score = 2.492; BH *p*-value = 1.000 × 10^–10^), IL-8 Signalling (*z*-score = 4.341; BH *p*-value = 5.495 × 10^–05^), Acute Phase Response Signalling (*z*-score = 4.523; BH *p*-value = 4.169 × 10^–04^) and IL-6 Signalling (*z*-score = 4.32; BH *p*-value = 9.120 × 10^–06^).

Furthermore, multiple pathways related to macrophages were upregulated in severe COVID-19 compared to moderate COVID-19. These included production of Nitric Oxide and Reactive Oxygen Species in Macrophages (*z*-score = 3.571; BH *p*-value = 1.689 × 10^–04^), Fcγ Receptor-mediated Phagocytosis in Macrophages and Monocytes (*z*-score = 2.959; BH *p*-value = 4.467 × 10^–05^) and Leukocyte Extravasation Signalling (*z*-score = 2.219; BH *p*-value = 8.710 × 10^–06^).

Other pathways identified as upregulated in severe COVID-19 included Cardiac Hypertrophy Signalling (*z*-score = 3.053; BH *p*-value = 1.318 × 10^–09^), Osteoarthritis Pathway (*z*-score = 2.546; BH *p*-value = 4.074 × 10^–06^) and Neuroinflammation Signalling Pathway (*z*-score = 2.557; BH *p*-value = 8.318 × 10^–07^). As observed in the severe *vs*. mild comparison, hypoxia related pathways were identified as enriched in severe COVID-19 including Hypoxia Signalling in the Cardiovascular System (*z*-score = 1.732; BH *p*-value = 4.571 × 10^–02^) and HIF1α Signalling (*z*-score = 2.109; BH *p*-value = 1.202 × 10^–07^).

Amongst the pathways downregulated in severe COVID-19 were various T cell pathways, including Th1 pathway (*z*-score = − 0.302; BH *p*-value = 1.000 × 10^–10^), T Cell Receptor Signalling (*z*-score − 7.553; BH *p*-value = 1.000 × 10^–06^), Systemic Lupus Erythematosus in T Cell Signalling Pathway (*z*-score: − 2.286; BH *p*-value = 4.467 × 10^–05^) and Calcium-induced T Lymphocyte Apoptosis (*z*-score: − 3.545; BH *p*-value = 2.951 × 10^–04^). PPAR signalling (*z*-score = − 1.0915; BH *p*-value = 4.571 × 10^–04^) and PPARα/RXRα activation (*z*-score = − 0.302; BH *p*-value = 3.548 × 10^–04^) were both downregulated in severe COVID-19, in addition to Antioxidant action of Vitamin C (*z*-score = − 2.828; BH *p*-value = 3.548 × 10^–02^) and FAT10 Cancer Signalling Pathway (*z*-score = − 1.807; BH *p*-value = 1.047 × 10^–02^).

### COVID-19 severity as an additive variable

**Figure 3:**
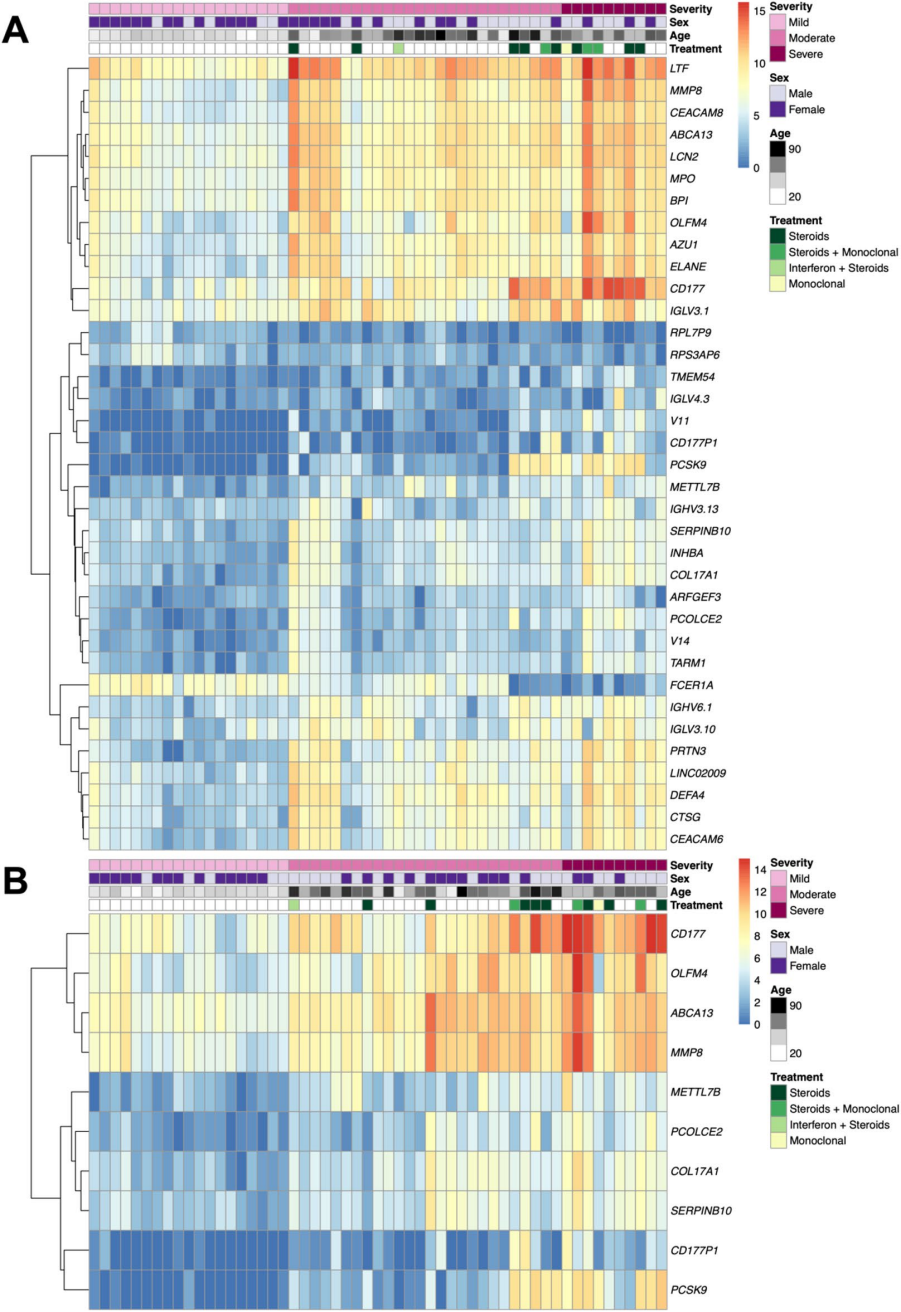
Heatmaps showing log-transformed expression values for (**A**) the 55 genes SDE with severity as an additive variable with absolute LFC values greater than 2 and B-H *p*-values < 0.0001 (**B**) the 10 genes SDE in all three pairwise severity comparisons in addition to the additive severity model with absolute LFC values greater than 2 and B-H *p*-values < 0.0001. Samples are ordered according to severity group. Age and sex were included as covariates in all differential expression analysis models.

The impacts of severity on the transcriptome were also explored with severity as an additive variable (Supplementary materials). 7413 genes were SDE with severity whilst accounting for immunomodulatory treatment, of which 55 had absolute LFC values greater than 2 and adjusted *p-*values < 0.0001 (Fig. 3A). The 55 genes are split into two broad clusters (Fig. 3A) with the 1st cluster containing multiple neutrophil-associated genes (including *LTF*, *MPO*, *BPI, ELANE*) and the 2nd cluster, with two sub-clusters, containing various immunoglobulin and B cell genes (e.g., *IGHV3-13, IGKV6-1, IGHV3-10)*. 307 genes were SDE in all three pairwise severity comparisons whilst controlling for immunomodulatory treatment in addition to displaying additive behaviour (Supplementary Table S6). Of these 307, 10 genes had absolute LFC values greater than 2 and adjusted *p-*values < 0.0001 for the additive analyses (Fig. 3B). Genes SDE in the additive model in addition to the pairwise comparisons show more granularity than those SDE just in the additive model as their levels differ between each severity category in a stepwise manner (Supplementary Fig. S6).

## Discussion

We have explored host whole-blood transcriptomes from COVID-19 patients with varying degrees of severity through differential expression and pathway analysis. We made pairwise comparisons between three different severity groups: mild, moderate, and severe. Severity analyses revealed major upregulation of genes and pathways related to the inflammatory immune response with increasing severity, with notable increases in genes and pathways related to neutrophil- and macrophage-mediated immunity accompanied by decreases in pathways related to T cell-mediated immunity.

We observed considerable transcriptomic differences between moderate COVID-19 individuals who received steroids to those who did not receive steroids, suggesting that this immunomodulatory treatment has a profound impact on the transcriptome. The widespread use of steroids in COVID-19 and the transcriptional disruption we observed in patients receiving steroids support the inclusion of immunomodulatory treatment in models related to COVID-19 transcriptomic analyses, in order to account for their impacts on the transcriptome.

Immune dysregulation has been extensively discussed as a contributing factor in the progression to severe COVID-19^5,8,36–38^. Infection with SARS-CoV-2 has been shown to induce a lower interferon response and an enhanced pro-inflammatory cytokine response in comparison to other viruses^39^. This pro-inflammatory cytokine response leads to the attraction of monocytes and neutrophils, the development of a cytokine storm and hyperinflammation, and is likely to contribute to COVID-19 severity^36,39^. Elevated levels of inflammatory cytokines and chemokines have been identified in plasma and serum from patients with increasing COVID-19 severity^37,40,41^. We observed a domination of immune system associated pathways upregulated with increasing severity, with inflammatory immune pathways consistently identified.

Comparison of the in silico estimates of immune cell proportions between the three severity groups revealed significant differences in all immune cell types except B cells. Lymphopenia was identified early in the pandemic as a key feature of COVID-19 severity^42,43^. Our results reflect this as the levels of CD4 and CD8 T cells and NK cells reduce with increasing severity, whilst pathway analysis contrasting severe COVID-19 to either moderate or mild COVID-19 also revealed downregulation of many T cell-related pathways.

Aschenbrenner et al.^18^ observed neutrophil-specific gene expression changes with increasing COVID-19 severity in whole blood. We found that the proportions of in silico neutrophil estimates increased with severity. Furthermore, we identified various pathways related to neutrophils that increased with severity, such as TREM1 signalling, Inhibition of Matrix Metalloproteases and the STAT3 pathway. TREM1 has been associated with neutrophil migration across airway epithelial cells and has been suggested to increase inflammation through neutrophil migration into the lung^44^. Matrix Metalloproteases (MMPs) are activated by neutrophil elastase and increased levels of multiple MMPs have been associated with increased COVID-19 severity^45,46^.

To add to observations of upregulated neutrophil-related pathways and increased in silico neutrophil estimates with severity, Fig. 3A shows a clear cluster of neutrophil-associated genes with high absolute LFC values that were SDE with additive severity. When the blood transcriptomic profiles of patients with mild COVID-19 were compared with the profiles from patients with moderate or severe COVID-19, *CEACAM8*, *MMP8*, *ELANE*, *LTF*, *CEACAM6* and *MPO* were consistently amongst the top SDE genes, with levels increasing with severity and high LFCs. *MMP8* was also found to have an additive effect with severity. *ELANE*, *MPO* and *PRTN3*, which are linked to neutrophil degranulation and NETosis, have been found to be significantly altered in naso-oropharyngeal samples of SARS-CoV-2 infected patients^47^. *ELANE*, *LTF*, *CEACAM8* and *MMP8* have been identified as being expressed in developing neutrophils, a novel cell subtype that was discovered through single-cell RNA sequencing of hospitalised COVID-19 patients, specifically identified in patients with acute respiratory distress syndrome (ARDS)^48,49^. *CEACAM6* has been identified as having high expression in Type II pneumocytes in COVID-19 patients, the cells targeted by SARS-CoV-2^50^, and it has been suggested that cross-talk between Type II pneumocytes and developing neutrophils in COVID-19 occurs via* CEACAM8*-*CEACAM6*^49^. It is possible that this cross-talk may promote differentiation of developing neutrophils, leading to further COVID-19 progression^49^.

Pathways related to macrophage activation were identified with increasing COVID-19 severity. Increased levels of macrophage inflammatory protein 1α and monocyte-derived FCN1 + macrophage cells have been detected in individuals with more severe COVID-19^51,52^. The observations of increased inflammatory immune pathways and increased neutrophil and macrophage activity were accompanied with downregulation of T cell related pathways with increasing severity, including upregulation and downregulation of Th2 and Th1 pathways, respectively, with increasing severity. These findings suggest that the balance between different immune cell types is a key component that influences severity of COVID-19.

When immune cell proportions were included in the pairwise severity models, there was a considerable reduction in the number of SDE genes identified (Fig. 2), with only one gene identified as SDE between severe and moderate COVID-19. This observation indicates that much of the transcriptomic differences between individuals with varying severity of COVID-19 are driven by different proportions of immune cells. Furthermore, a large proportion of the pathways identified as enriched with severity were related to functions of different immune cells, highlighting that they play a major role in the pathogenesis of severe COVID-19.

Other pathways of interest include pathways related to hypoxia that were enriched with increasing severity. Hypoxia is a primary feature and major cause of mortality amongst patients with severe COVID-19^53^. Various pathways related to protein production and DNA/protein damage were identified as downregulated with increasing COVID-19 severity. SARS-CoV-2 has been shown to cause major disruption to host protein production, for example viral protein NSP1 has been shown to bind to the host 40S ribosomal subunit resulting in mRNA translation shut down^54,55^. Furthermore, coronaviruses have also been shown to use DNA damage to induce cell cycle arrest^56^. Damaged DNA can lead to accumulation of nuclear DNA in the cytoplasm which triggers innate immune responses^57^.

Neutrophilia and neutrophil activation are usually markers of bacterial infection and are uncommon in most uncomplicated viral infections. In view of the association between increasing severity of COVID-19 with increased neutrophil counts, and expression of neutrophil genes including those involved in degranulation, NETosis, and neutrophil-mediated tissue injury, a key question is what mechanisms are responsible for the shift from the normal “viral” response in mild COVID-19, to the severe inflammatory process involving neutrophils in severe disease. The timing of the inflammatory phase of COVID-19 that usually occurs in the second week of illness^58,59^, together with the increased expression of immunoglobulin genes that we observed (e.g., *IGKV1D-13, IGHV3-43, IGLV4-3*, *IGLV3-16*, *IGLV3-10*) may suggest that immunoglobulin, directed at either viral or modified self-antigens, may be involved in neutrophil and macrophage activation through Fc-gamma receptors or complement mediated activation, genes related to which are upregulated in severe disease (e.g., *FCGR2A, FCGR3B, FCGR3A, CR1, C3AR1, C5AR1*). Neutrophil activation and neutrophil-mediated tissue injury may be a promising target for therapeutic interventions.

This study has identified many genes and pathways that are associated with differing COVID-19 severity, amongst which there could be some promising novel targets for immunomodulatory therapies for preventing severe COVID-19. As such, the genes and pathways identified here warrant further investigation.

### Limitations

COVID-19 severity is highly influenced by age and sex at birth which leads to major confounding between COVID-19 and these two variables. Although we controlled for these confounders (by including sex, age, and the interaction between age and severity) when exploring transcriptomic changes with severity, it is possible that we may have (a) failed to identify key drivers of severity as they are confounded with age or sex, (b) inadvertently included spurious genes that are really driven by age or sex rather than severity. Furthermore, patients included in this study had various comorbidities, with the frequency of some comorbidities higher in severe patients (obesity, smoking, endocrine conditions). These comorbidities could influence gene expression changes, introducing another confounder into the analysis. Due to lack of detailed documentation for all subjects for these comorbidities, it was not possible to correct for the variance introduced as was done with age and sex. The sample sizes in our analyses are modest for some severity groups. For example, in the severe COVID-19 group, only 10 samples could be included as the rest were excluded due to concomitant bacterial infection. These samples were excluded because coinfections were likely to have had profound transcriptional impacts and may have masked the genuine SARS-CoV-2 signal.

In addition, we were not able to correct for all types of immunomodulatory treatment. Specifically, macrolides which are known immunomodulatory antibiotics, were administered to all moderate and severe COVID-19 patients and to none of the mild COVID-19 patients (Table 1). As a result, we were unable to include macrolides in our models accounting for immunomodulatory treatment since the model would have been in full rank, i.e., impossible to disentangle the severity and macrolides.

### Conclusion

We have explored the transcriptomic impact of SARS-CoV-2 infection through evaluating the transcriptomic differences between individuals with varying levels of COVID-19 severity. We have observed considerable transcriptomic perturbation which offers insights into the host factors that influence development of severe COVID-19. Upregulation of inflammatory immune pathways was observed with increasing severity, with multiple neutrophil, macrophage and immunoglobulin-associated genes and pathways identified, suggesting that increased COVID-19 severity may be mediated in part by neutrophil activation, which may be related to production of immunoglobulin as acquired immunity develops. Furthermore, we have discovered that administration of steroids leads to profound changes in the whole blood transcriptome of individuals with similar COVID-19 severity, highlighting the importance of considering the effects of treatment in future COVID-19 transcriptomic studies.

## Supplementary Information

Supplementary Information 1.

Supplementary Information 2.

Supplementary Information 3.

Supplementary Information 4.

Supplementary Information 5.

Supplementary Information 6.

Supplementary Information 7.

## Data Availability

Gene counts and sample metadata are available on ArrayExpress under the accession E-MTAB-10926. Code used in the analyses can be found at: https://github.com/PIDBG/sarscov2_transcriptomics/tree/main/severity_analysis.
