# Supplementary material for: Characterisation of the blood RNA host response underpinning severity in COVID-19 patients
Source: Sci Rep. 2022 Jul 17;12:12216. doi: 10.1038/s41598-022-15547-2 (PMC9288817; doi:10.1038/s41598-022-15547-2)
Supplement: Supplementary file 7 — Supplementary Information 7. [file 41598_2022_15547_MOESM7_ESM.docx]

*Legend for supplementary files:*

**Supplementary file 1 (File_S1_Steroids.xlsx)** File contains list of genes significantly differentially expressed (SDE) between moderate COVID-19 patients who did receive steroids *vs*. moderate COVID-19 patients who did not receive steroids.

**Supplementary file 2 (File_S2_Moderate_vs_Mild_COVID19.xlsx)** File contains lists of genes SDE and pathways enriched between moderate COVID-19 *vs*. mild COVID-19 with including either 1) immunomodulatory treatment in the model, or 2) immune cell proportions in the model.

**Supplementary file 3 (File_S3_Severe_vs_Mild_COVID19.xlsx)** File contains lists of genes SDE and pathways enriched between severe COVID-19 *vs*. mild COVID-19 with including either 1) immunomodulatory treatment in the model, or 2) immune cell proportions in the model.

**Supplementary file 4 (File_S4_Severe_vs_Moderate_COVID19.xlsx)** File contains lists of genes SDE and pathways enriched between severe COVID-19 *vs*. moderate COVID-19 with including either 1) immunomodulatory treatment in the model, or 2) immune cell proportions in the model.

**Supplementary file 5 (File_S5_Additive_Severity.xlsx)** File contains lists of genes SDE and pathways enriched with increasing COVID-19 severity (as an additive variable) with including either 1) immunomodulatory treatment in the model, or 2) immune cell proportions in the model.

**Supplementary file 6 (File_S6_Hospitalised_vs_Non_Hospitalised.xlsx)**

1. ***Supplementary Methods***

*DESeq2*

Differential expression analysis was performed using DESeq2 [1]. DESeq2 fits a generalised linear regression model (GLM) for each gene, modelling the changes in gene expression according to covariates included in the design matrix. The design matrix not only includes the variable of interest, but can also include factors to control. DESeq2 uses Empirical Bayes and is able to pool information across genes to allow for accurate dispersion estimates.

For the pairwise comparisons, two models were used with one model accounting for immunomodulatory treatment and the other model accounting for immune cell proportions. Both models also accounted for age and sex. By including all samples (mild, moderate, severe COVID-19) in the models, the impact of covariates can be modelled across all samples, leading to more reliable estimates. As such, it is possible to correct for confounding introduced by covariates that may not impact all groups of samples, for example it is possible to account for immunomodulatory treatment in mild *vs*. severe COVID-19 and mild *vs*. moderate COVID-19 despite none of the mild COVID-19 patients receiving immunomodulatory treatment.

*Ingenuity Pathway Analysis*

Pathway analysis was performed on the SDE genes using Ingenuity Pathway Analysis (IPA; QIAGEN Inc., <https://www.qiagenbioinformatics.com/products/ingenuity-pathway-analysis>). For each pathway identified, IPA returns a *p*-value in addition to a z-score. The z-score is computed by comparing the direction of the expression pattern of genes included in the analysis (i.e., through using the log2 fold change) with the expected direction of the same molecules from the Ingenuity Knowledge Base, a large structured collection of observations in various experimental contexts with findings manually curated from the biomedical literature or integrated from third-party databases [2]. Z-scores are used to indicate the direction of change of each pathway and the degree of change. Higher absolute z-scores indicate a larger degree of change between the groups, with positive and negative z-scores indicating activation and repression of a pathway, respectively. Z-scores and p-values are independent metrics, and it is possible to have a significant adjusted p-value but z-score=0. In this instance, IPA can detect that the pathway is significantly enriched, but it is not able to determine the directionality of the pathway, i.e., whether it is activated or repressed

A simplified z-score can be calculated by:

$$z= \frac{x}{\sigma_{x}}=\frac{\Sigma_{i}x_{i}}{\sqrt{N}}=\frac{N_{+}-N_{-}}{\sqrt{N}}$$

where $N_{+}$ are the genes whose up/down direction agrees with the literature, and $N_{-}$ are the genes whose up/down direction disagree with the literature [2].

*GAMLSS*

An alternative approach to differential expression analysis was explored using GAMLSS (generalised additive models for location, scale and shape) [3]. GAMLSS was used since it enables the user to control the modelling of variability in the dataset. GAMLSS was ran twice for each pairwise comparison with either immunomodulatory treatment included in the model in addition to age and sex, or immune cell proportions included in the model in addition to age and sex. The GAMLSS and DESeq2 models were contrasted against each other, generating cross plots and calculating how many genes were SDE (B-H *p*-value < 0.05) from both methods.

*Additive model*

Severity was explored as a numerical variable in an additive model with 0, 1 and 2 values indicating mild, moderate, and severe cases, respectively, to identify genes that are significantly differentially expressed (SDE) across the three severity groups sequentially. The analysis was repeated twice with different model designs. The first model design aimed to account for transcriptomic differences induced by immunomodulatory treatments. This model included parameters representing whether the patients received tocilizumab, steroids or interferon treatments, in addition to sex, age, severity. The second model design accounted for transcriptomic differences induced by different proportions of immune cells. The immune cell proportions accounted for included: monocytes, neutrophils, B cells (the sum of naïve and memory B cells and plasma cell proportions), CD4 T cells, (the sum of the proportions of naïve CD4 T cells, resting and activated memory CD4 T cells, follicular helper T cells and regulatory T cells), CD8 T cells and natural killer cells (the sum of resting and activated natural killer [NK] cell proportions). In addition to the immune cell proportions, this model included sex, age, severity. Pathway analysis using IPA was performed on the SDE genes. Genes SDE with age were also identified through using a DESeq2 model with age, severity (as an additive variable) the interaction between and severity and sex. This model designed to identify genes SDE with age that could be excluded due to confounding between age and severity.

*Hospitalised vs. Non-Hospitalised sample comparison*

Samples from hospitalised COVID-19 patients (moderate and severe) were contrasted against samples from non-hospitalised COVID-19 patients (mild) using DESeq2 with age, sex and immunomodulatory treatment included in the design matrix. Genes identified as SDE were contrasted against the other models evaluated.

1. ***Supplementary Results***
   1. *In silico immune cell proportion estimates*

The impact of age and sex on the levels of *in silico* immune cell proportion estimates were explored to determine whether the changes observed in immune cell proportions across COVID-19 severity groups were driven by either of these factors in addition to COVID-19 severity.

Supplementary Figure 3 shows the immune cell proportions across COVID-19 severity groups split according to sex with Benjamini-Hochberg *p*-values from two-sided Mann-Whitney-Wilcoxon test contrasting males to females shown on the plots. Males and females with severe COVID-19 showed significantly different levels of natural killer (NK) cells (*p*-value: 0.03; Supplementary Figure 3) with levels increasing in females, however this trend was not observed in NK cells for either mild or moderate COVID-19. All other comparisons were not significantly different between males and females, indicating that the differences in immune cell proportions displayed in Supplementary Figure 2 are not driven by sex.

Supplementary Figure 4 shows the relationship between immune cell proportions and age across COVID-19 severity groups. The relationship between age and each immune cell type was tested for each severity group using linear regression models, and the Benjamini-Hochberg *p*-values from the linear regression models are shown in Supplementary Table 1. The only comparisons that reached significance (B-H *p*-value < 0.05) were CD4 T cells (B-H *p*-value: 0.003) and neutrophils (B-H *p*-value: 0.005) in moderate COVID-19, with levels decreasing and increasing with age, respectively. These trends were not observed in the other severity groups for the respective immune cell proportions. Indeed, for severe COVID-19 samples, the proportion of CD4 T cells appeared to increase with age, unlike in moderate COVID-19. Furthermore, there was a clear distinction in the CD4 T cells and neutrophil proportions between the severe COVID-19 samples and age-matched moderate COVID-19 samples with almost completely distinct confidence intervals (Supplementary Figure 4). Overall, these results indicate that age is not driving the immune cell proportion changes observed in Supplementary Figure 2.

- 1. *The effect of immunomodulatory treatment on COVID-19 patients’ blood transcriptome*

The impacts of steroid administration on the transcriptome were explored through contrasting moderate COVID-19 patients who received steroids (*n*=6) to moderate COVID-19 patients who did not receive steroids (*n*=19). IPA identified no significant pathways from the SDE genes. Full results in File_S1_Steroids.xlsx.

- 1. *Transcriptomic differences between different COVID-19 severity groups*

*Moderate COVID-19 vs*. *Mild COVID-19*

Full lists of SDE genes and pathways are in Supplementary File 2 (File_S2_Moderate_vs_Mild_COVID19.xlsx). IPA identified 24 significant pathways (Supplementary Table 3) from the list of genes SDE between moderate and mild COVID-19 whilst accounting for immunomodulatory treatment (*n*=1,547), with 9 and 12 pathways up and downregulated, respectively. IPA identified two significant pathways (EIF2 Signalling: *z*-score=-2.53, B-H *p*-value=1.288×10^-03^; Regulation of eIF4 and p70S6K Signalling: B-H *p*-value=1.950×10^-02^) from the list of genes SDE between moderate and mild COVID-19 whilst accounting for immune cell proportions (*n*=488).

*Severe COVID-19 vs. Mild COVID-19*

Full lists of SDE genes and pathways are in Supplementary File 3 (File_S3_Severe_vs_Mild_COVID19.xlsx). IPA identified 68 significant pathways (Supplementary Table 4) from the list of genes SDE between severe COVID-19 and mild COVID-19 whilst accounting for immunomodulatory treatment (*n*=7,343), with 33 and 19 pathways upregulated and downregulated, respectively. When immune cell proportions were included in the model instead of immunomodulatory treatment, IPA identified one significant pathway (Airway Pathology in Chronic Obstructive Pulmonary Disease, B-H *p*-value=4.571×10^-04^) from the list of genes SDE (*n*=94).

*Severe COVID-19 vs*. *Moderate COVID-19*

Full lists of SDE genes and pathways are in Supplementary File 4 (File_S4_Severe_vs_Moderate_COVID19.xlsx). IPA identified 260 significant pathways (Supplementary Table 5) from the list of genes SDE between severe COVID-19 and moderate COVID-19 whilst accounting for immunomodulatory treatment (*n*=8,971) with 179 and 23 pathways up and down regulated, respectively. When immune cell proportions were included in the model instead of immunomodulatory treatment, one SDE genes was identified (*NGRF*) so pathway analysis was not performed.

- 1. *Using GAMLSS to explore transcriptomic differences with COVID-19 severity*

An alternative approach to differential expression analysis, GAMLSS [3], was used for the pairwise severity models and the results were compared between statistical approaches. GAMLSS is a family of regression models that has been used before for differential expression analysis [4, 5] with the added benefit that variances can be controlled according to the distributions of samples in the analysis.

A total of 1,438 genes were identified as SDE between moderate *vs*. mild COVID-19 whilst accounting for immunomodulatory treatment by both DESeq2 and GAMLSS with concordant directions of change (Supplementary Figure 5A). GAMLSS and DESeq2 identified a further 847 and 109 SDE genes, respectively. For the models contrasting moderate *vs*. mild COVID-19 that included immune cell proportions, 470 genes were identified as SDE with concordant directions of change by DESeq2 and GAMLSS (Supplementary Figure 5B). GAMLSS and DESeq2 identified a further 1,933 and 18 SDE genes, respectively.

A total of 5,488 genes were identified as SDE between severe *vs*. mild COVID-19 whilst accounting for immunomodulatory treatment by both DESeq2 and GAMLSS with concordant directions of change (Supplementary Figure 5C). GAMLSS and DESeq2 identified a further 2,956 and 1,855 SDE genes, respectively. For the models contrasting severe *vs*. mild COVID-19 that included immune cell proportions, 42 genes were identified as SDE with concordant directions of change by DESeq2 and GAMLSS (Supplementary Figure 5D). GAMLSS and DESeq2 identified a further 2,703 and 52 SDE genes, respectively.

A total of 6,145 genes were identified as SDE between severe *vs*. moderate COVID-19 whilst accounting for immunomodulatory treatment by both DESeq2 and GAMLSS with concordant directions of change (Supplementary Figure 5E). GAMLSS and DESeq2 identified a further 686 and 2,810 SDE genes, respectively. For the models contrasting severe vs. mild COVID-19 that included immune cell proportions, GAMLSS identified 27 SDE genes, however none of them were the single gene identified by DESeq2 for this model.

- 1. *Additive Severity Model*

In addition to exploring the transcriptomic differences between the pairwise severity groups, we also explored severity as an additive variable to attempt to identify genes that are SDE across all three groups sequentially. The full lists of genes and pathways are in Supplementary File 5 (File_S5_Additive_Severity.xlsx). The models exploring severity as an additive variable were ran twice; once with immunomodulatory treatment variables included in the model, and once with immune cell proportions included in the model. When the immunomodulatory treatments were accounted for the in DESeq2 model, there were 7,414 genes SDE (adjusted *p*-value < 0.05) with severity. 3,626 genes were over-expressed with severity and 3,788 genes were under-expressed with increasing severity. When the cell proportions were accounted for the in DESeq2 model, there were 88 genes SDE (adjusted *p*-value < 0.05) with severity. 74 genes were over-expressed with severity and 14 genes were under-expressed with increasing severity. Whilst 82 genes were SDE in both comparisons (Supplementary Fig. 6), the treatment and immune cell corrections revealed 7,330 and 6 additional SDE genes, respectively.

IPA pathway analyses were performed. When immunomodulatory treatment was included in the model, 123 significant pathways were identified by IPA with 84 and 19 pathways increasing and decreasing with severity, respectively (Supplementary Table 6). When cell proportions were included in the model, one pathway was identified by IPA: Airway Pathology in Chronic Obstructive Pulmonary Disease (B-H *p*-value=3.89×10^-04^).

307 genes were identified as having additive behaviour in addition to being SDE in pairwise analyses whilst correcting for immunomodulatory treatment (Supplementary Table 7). Of the 307 genes, all log_2_ fold-change directions were concordant with 96 and 211 genes increasing and decreasing with severity, respectively.

- 1. *Contrasting hospitalised to non-hospitalised patients*

Genes SDE between samples from patients hospitalised (moderate, severe) *vs*. samples from patients non-hospitalised (mild) were identified by DESeq2 with a model accounting for age, sex and immunomodulatory treatment. A total of 1,733 genes were SDE, with 784 and 949 increasing and decreasing, respectively, in hospitalised *vs*. non-hospitalised patients. Of the 1,733 genes, 1,397 were also SDE between moderate COVID-19 *vs*. mild COVID-19, 1,484 were also SDE between severe COVID-19 *vs*. mild COVID-19, and 506 were also SDE between severe COVID-19 *vs*. moderate COVID-19.

Six genes were SDE between hospitalised *vs*. non-hospitalised COVID-19 that were not SDE in any of the pairwise comparisons, nor in the additive models. These genes were: IGKV2D-29 (B-H *p*-value = 5.21x10^-4^, LFC = 2.951); MIR8064 (B-H *p*-value = 0.041, LFC = 0.666); OR52N4 (B-H *p*-value = 0.042; LFC = 0.958); SNAPC4 (B-H *p*-value = 0.044, LFC = 0.303); IGHV2-26 (B-H *p*-value = 0.049, LFC = 2.139); CENPM (B-H *p*-value = 0.049, LFC = 0.874). The full list of SDE genes is found in Supplementary File 6.

- 1. *Identification of genes SDE with age*

We used DESeq2 to identify genes SDE with age in COVID-19 patients in our dataset. The model included sex, age, severity, and the interaction between age and severity. We did this to see whether there would be genes that could be SDE with severity but were not identified due to their associations with age. 25 genes were SDE with age (Supplementary Table 8).

****Supplementary Figure 1 Principal component analysis (PCA) plots for the samples used in the analyses. Points, which represent samples, are coloured by disease severity (A), sex (B) and age (C).


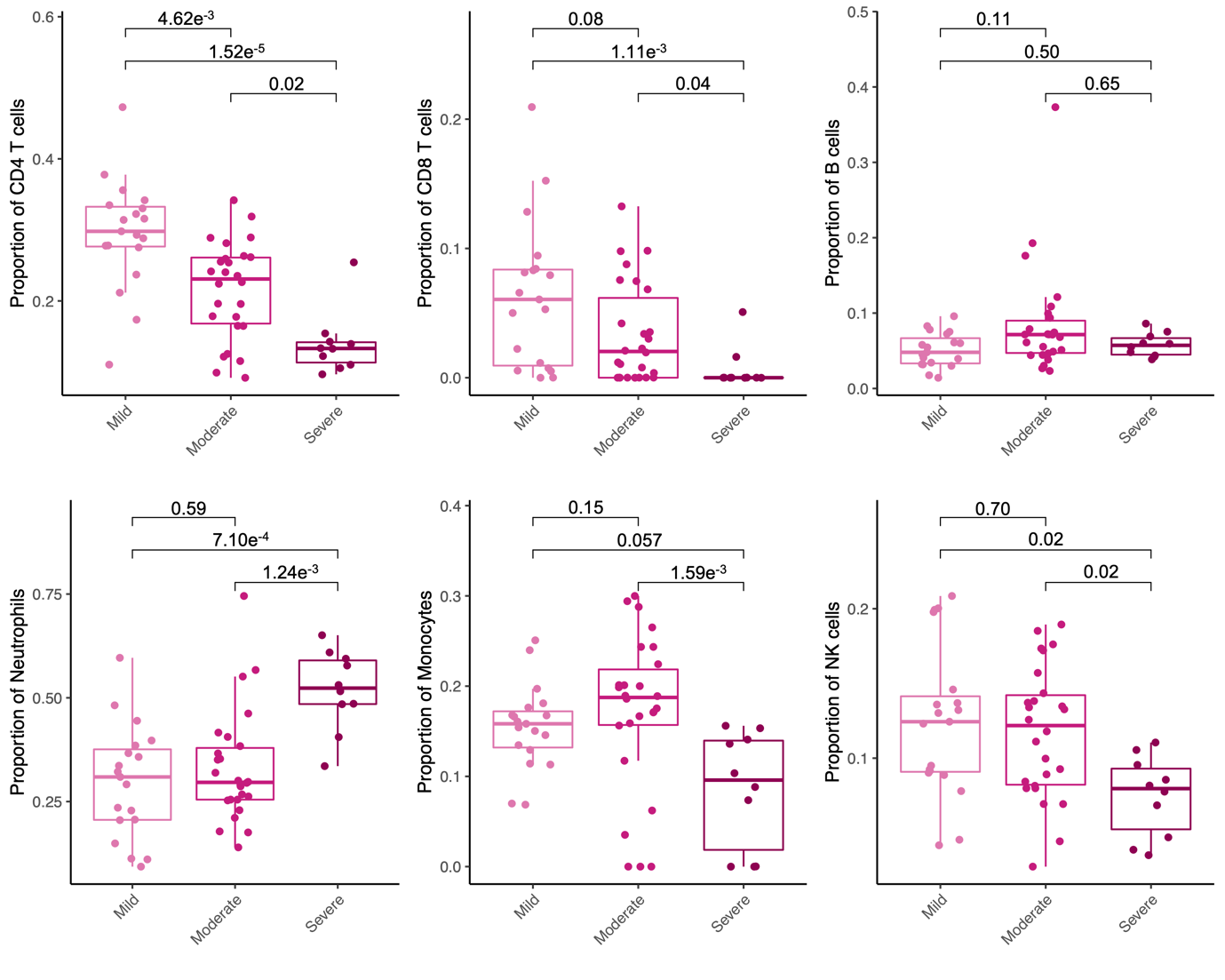


Supplementary Figure 2 *In silico* predicted immune cell proportions for COVID-19 samples across severity groups. Immune cell proportions were estimated using CIBERSORTx [6]. B cells are the sum of naïve and memory B cells and plasma cell proportions, CD4 T cells are the sum of naïve CD4 T cells, resting and activated memory CD4 T cells, follicular helper T cells and regulatory T cells proportions, and natural killer cells are the sum of resting and activated NK cell proportions. *P*-values are from Dunn’s test with Benjamini-Hochberg correction.

Supplementary Figure 3 *In silico* predicted immune cell proportions for COVID-19 samples across severity groups split according to sex. Immune cell proportions were estimated using CIBERSORTx [6]. B cells are the sum of naïve and memory B cells and plasma cell proportions, CD4 T cells are the sum of naïve CD4 T cells, resting and activated memory CD4 T cells, follicular helper T cells and regulatory T cells proportions, and natural killer cells are the sum of resting and activated NK cell proportions. *P*-values are calculated using two-sided Mann-Whitney-Wilcoxon with Benjamini-Hochberg correction.

Supplementary Figure 4 *In silico* predicted immune cell proportions levels *vs*. age with samples and linear regression trend lines coloured according to COVID-19 severity. Immune cell proportions were estimated using CIBERSORTx [6]. B cells are the sum of naïve and memory B cells and plasma cell proportions, CD4 T cells are the sum of naïve CD4 T cells, resting and activated memory CD4 T cells, follicular helper T cells and regulatory T cells proportions, and natural killer cells are the sum of resting and activated NK cell proportions. Grey bands represent 95% confidence intervals.


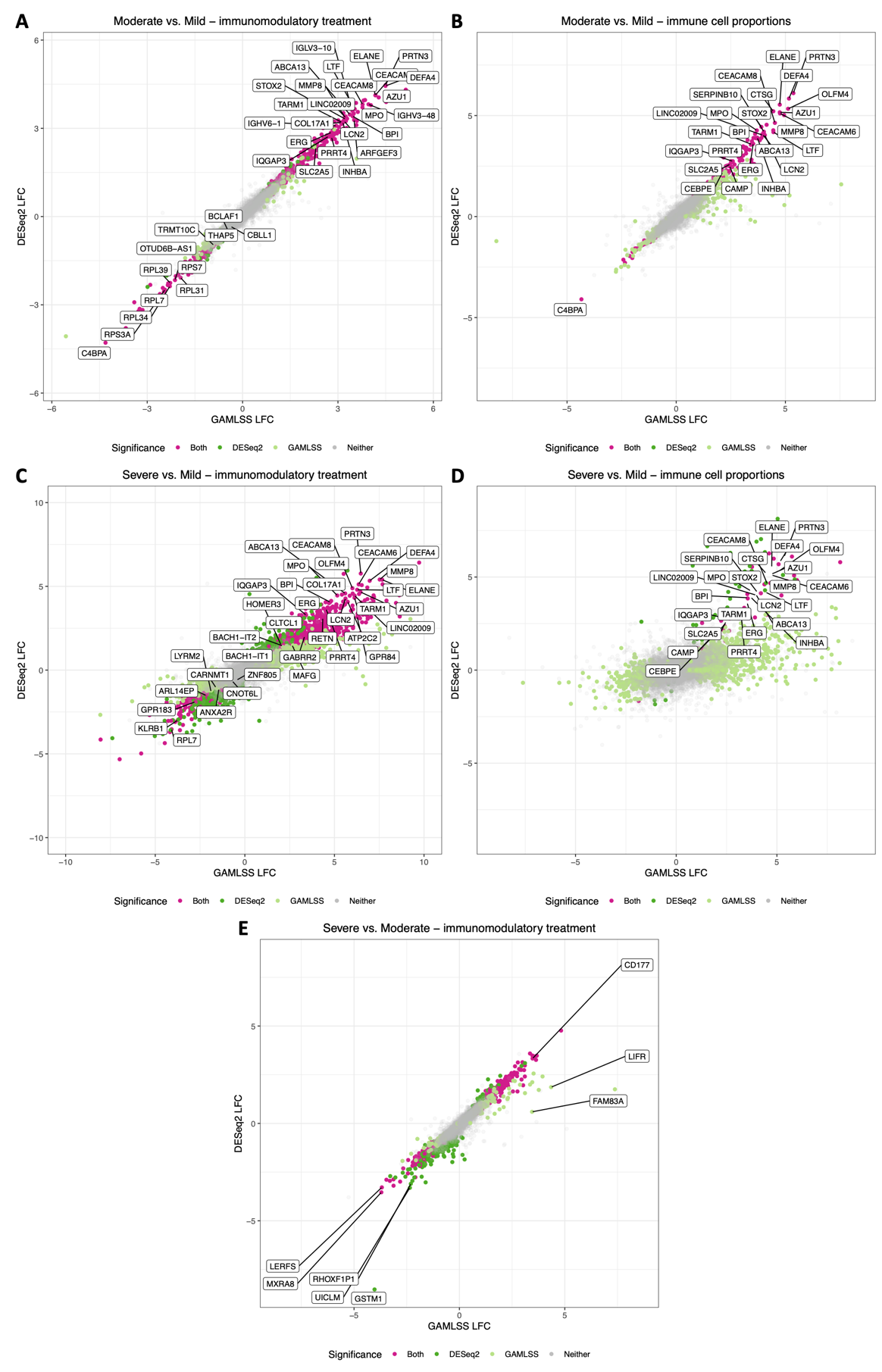


Supplementary Figure 5 Cross plots showing the log_2_ fold change (LFC) values of genes for three severity groups (**A-B**: moderate *vs.* mild; **C-D**: severe *vs.* mild; **E** severe *vs.* moderate), with models accounting for either immunomodulatory treatment (**A, C, E**) or immune cell proportions (**B, D**). The plots show how LFC values differ according to differential expression analysis was performed by DESeq2 or GAMLSS. Dark pink points are genes that were identified as SDE by both methods, whilst dark green and light green points are genes identified as SDE by DESeq2 or GAMLSS, respectively. NS = not significant.

**Supplementary Figure 6** Cross plot showing the log2 fold change (LFC) values of genes with severity as an additive variable and how LFC values differ according to whether immune cell proportions (x-axis) or immunomodulatory treatments (y-axis) are included in the model. Red points are genes that are were SDE in both models, whilst orange and green points are genes SDE in the cell correction and treatment correction models, respectively. NS = not significant.

**Supplementary Figure 7** Boxplots showing the normalised gene counts for the top genes SDE in the additive severity analysis in addition to each pairwise comparison from the DESeq2 models including age, sex and immunomodulatory treatment. These genes had absolute LFC values greater than 2 and adjusted p-values < 0.0001 in the additive DESeq2 model and are also shown in Fig. 3B.

**Supplementary Table 1** *p*-values obtained from linear regression models contrasting age to *in silico* immune cell proportions within each of the COVID-19 severity groups. *p*-values are Benjamini-Hochberg adjusted.

| **In silico immune cell proportions** | **Mild COVID-19 p-value** | **Moderate COVID-19 p-value** | **Severe COVID-19 p-value** |
| --- | --- | --- | --- |
| CD4 T cells | 0.374 | 0.003 | 0.241 |
| CD8 T cells | 0.746 | 0.746 | 0.746 |
| B cells | 0.995 | 0.995 | 0.995 |
| Neutrophils | 0.380 | 0.005 | 0.645 |
| Monocytes | 0.168 | 0.633 | 0.922 |
| Natural Killer cells | 0.812 | 0.055 | 0.812 |

**Supplementary Table 2** Clinical and demographic data for the samples included in the analysis contrasting moderate COVID-19 patients who received steroids (*n*=6) to moderate COVID-19 patients who did not receive steroids (*n*=19).

|  | **Administered steroids (n=6)** | **Not administered steroids (n=19)** |
| --- | --- | --- |
| **Demographic features** | | |
| Sex (female) | 3 (50%) | 9 (47%) |
| Age (median) | 73 (72-79) | 68 (56-74) |
| <50 years | 0 (0%) | 4 (21%) |
| 50-70 years | 0 (0%) | 10 (53%) |
| >70 years | 6 (100%) | 5 (26%) |
| Ethnicity (South European) | 6 (100%) | 18 (95%) |
| **Comorbidities** |  |  |
| Endocrine | 4 (66.7%) | 10 (52.6%) |
| Obesity | 3 (50%) | 9 (47%) |
| Hypertension | 3 (50%) | 3 (15.8%) |
| Cardiac | 2 (33.3%) | 5 (26%) |
| Gastrointestinal | 1 (16.7%) | 4 (21%) |
| Smoking | 1 (16.7%) | 3 (15.8%) |
| Pulmonary | 1 (16.7%) | 6 (66.7%) |
| **Timeline** | | |
| Days since symptom onset (median, IQR) | 13.5 (13-14.8) | 13 (11.5-16.5) |
| **Presenting symptoms** | | |
| Fever | 5 (83%) | 16 (84%) |
| Respiratory | 6 (100%) | 17 (89% |
| Cardiac | 0 (0%) | 1 (5%) |
| Gastrointestinal | 1 (16.7%) | 8 (42%) |
| Musculoskeletal | 3 (50%) | 13 (68%) |
| Sensory (ageusia, anosmia) | 2 (33.3%) | 6 (66.7%) |
| Headache | 0 (0%) | 4 (21%) |
| Ill appearance | 0 (0%) | 2 (10.5%) |
| **Treatment** |  |  |
| Macrolides (azithromycin) | 6 (100%) | 19 (100%) |
| Cephalosporins (ceftriaxone, cefeprime) | 3 (50%) | 11 (57.9%) |
| Antiviral (lopinavir-ritonavir) | 6 (100%) | 15 (78.9%) |
| Antimalarial (hydroxychloroquine) | 6 (100%) | 18 (94.7%) |
| **Clinical investigations and interventions** | | |
| White cells (10^9^/L) – mean (IQR) | 8 (5-14) | 6 (5.4-6.4) |
| Neutrophils (10^9^/L) – mean (IQR) | 5.5 (3-11) | 3.3 (2.6-4.0) |
| Lymphocytes (10^9^/L) – mean (IQR) | 1.3 (0.6-2.7) | 1.5 (1.2-1.9) |
| Monocytes (10^9^/L) – mean (IQR) | 0.2 (0.2-0.5) | 0.4 (0.3-0.5) |
| Fibrinogen (mg/dL)- mean (IQR) | 439 (393-500) | 485 (415-500) |
| D-dimer (ng/mL)- mean (IQR) | 666 (580-660) | 890 (452-1306) |
| CRP (mg/L)- mean (IQR) | 11 (6.4-17.6) | 25 (19-36) |
| Ferritin (μg/L)- mean (IQR) | 925 (624-1355) | 317 (152-518) |
| Oxygen treatment | 6 (100%) | 14 (74%) |

**Supplementary Table 3** The top 15 pathways (from a total of 24 significant pathways) identified by IPA from the genes SDE between moderate COVID-19 *vs*. mild COVID-19 (B-H p-value < 0.05) whilst correcting for immunomodulatory treatment. Positive and negative Z-scores indicate pathway upregulation and downregulation, respectively, in moderate COVID-19 *vs*. mild COVID-19.

| **Ingenuity Canonical Pathways** | **B-H p-value** | **Z-score** |
| --- | --- | --- |
| EIF2 Signaling | 5.012×10^-29^ | -5.778 |
| Regulation of eIF4 and p70S6K Signaling | 1.000×10^-13^ | -1 |
| mTOR Signaling | 4.467×10^-08^ | -0.632 |
| Coronavirus Pathogenesis Pathway | 5.248×10^-07^ | 2.744 |
| Kinetochore Metaphase Signaling Pathway | 1.820×10^-05^ | 0 |
| Mitotic Roles of Polo-Like Kinase | 2.754×10^-05^ | 0.816 |
| Oxidative Phosphorylation | 1.514×10^-03^ | -4.243 |
| Unfolded protein response | 1.514×10^-03^ | -1 |
| Mitochondrial Dysfunction | 2.692×10^-03^ | NA |
| Cell Cycle Regulation by BTG Family Proteins | 5.888×10^-03^ | NA |
| Sirtuin Signaling Pathway | 5.888×10^-03^ | 0.218 |
| Huntington's Disease Signaling | 6.026×10^-03^ | 0.302 |
| Role of CHK Proteins in Cell Cycle Checkpoint Control | 7.943×10^-03^ | -2.828 |
| ATM Signaling | 7.943×10^-03^ | 0 |
| Spliceosomal Cycle | 8.318×10^-03^ | -3.162 |

**Supplementary Table 4** The top 15 (from a total of 68 significant pathways) identified by IPA from the genes SDE between severe COVID-19 *vs*. mild COVID-19 (B-H p-value < 0.05) whilst correcting for immunomodulatory treatment. Positive and negative Z-scores indicate pathway upregulation and downregulation, respectively, in severe COVID-19 *vs*. mild COVID-19.

| **Ingenuity Canonical Pathways** | **B-H p-value** | **Z-score** |
| --- | --- | --- |
| EIF2 Signaling | 1.259×10^-19^ | -6.14 |
| Regulation of eIF4 and p70S6K Signaling | 1.230×10^-08^ | -0.728 |
| mTOR Signaling | 9.333×10^-06^ | -1 |
| Coronavirus Pathogenesis Pathway | 9.333×10^-06^ | 3.667 |
| Th1 and Th2 Activation Pathway | 1.820×10^-05^ | NA |
| Th2 Pathway | 1.549×10^-04^ | 1.372 |
| Th1 Pathway | 7.413×10^-04^ | -0.949 |
| Natural Killer Cell Signaling | 7.413×10^-04^ | 0 |
| 4-1BB Signaling in T Lymphocytes | 9.120×10^-04^ | 0.728 |
| Role of PKR in Interferon Induction and Antiviral Response | 9.120×10^-04^ | 1.54 |
| B Cell Receptor Signaling | 1.479×10^-03^ | 1.408 |
| RANK Signaling in Osteoclasts | 2.754×10^-03^ | 1.715 |
| Role of CHK Proteins in Cell Cycle Checkpoint Control | 3.890×10^-03^ | -2.236 |
| Unfolded protein response | 3.890×10^-03^ | 1.043 |
| HIF1α Signaling | 3.890×10^-03^ | 2.255 |

**Supplementary Table 5** The top 15 pathways (from a total of 260 significant pathways) by IPA from the genes SDE between severe COVID-19 *vs*. moderate COVID-19 (B-H p-value < 0.05) whilst correcting for immunomodulatory treatment. Positive and negative Z-scores indicate pathway upregulation and downregulation, respectively, in severe COVID-19 *vs*. moderate COVID-19.

| **Ingenuity Canonical Pathways** | **B-H p-value** | **Z-score** |
| --- | --- | --- |
| Th1 and Th2 Activation Pathway | 1.995×10^-12^ | NA |
| Th1 Pathway | 1.000×10^-10^ | -0.302 |
| Natural Killer Cell Signaling | 1.000×10^-10^ | 2.492 |
| Th2 Pathway | 2.291×10^-10^ | 1.441 |
| STAT3 Pathway | 3.388×10^-10^ | 1.808 |
| TREM1 Signaling | 6.310×10^-10^ | 4.218 |
| Cardiac Hypertrophy Signaling (Enhanced) | 1.318×10^-09^ | 3.053 |
| Hepatic Fibrosis Signaling Pathway | 1.023×10^-08^ | 2.376 |
| Role of Macrophages, Fibroblasts and Endothelial Cells in Rheumatoid Arthritis | 3.631×10^-08^ | NA |
| NF-κB Signaling | 1.072×10^-07^ | 2.066 |
| HIF1α Signaling | 1.202×10^-07^ | 2.109 |
| Tec Kinase Signaling | 1.202×10^-07^ | 2.111 |
| Axonal Guidance Signaling | 1.202×10^-07^ | NA |
| PI3K/AKT Signaling | 2.512×10^-07^ | 1.095 |
| HGF Signaling | 2.512×10^-07^ | 2.744 |

**Supplementary Table 6** The top 15 pathways (from a total of 123 significant pathways) by IPA from the genes SDE with severity as an additive variable whilst correcting for immunomodulatory treatment. Positive and negative Z-scores indicate pathway upregulation and downregulation, respectively, with increasing COVID-19 severity.

| **Ingenuity Canonical Pathways** | **B-H p-value** | **Z-score** |
| --- | --- | --- |
| EIF2 Signaling | 2.512×10^-22^ | -5.421 |
| Regulation of eIF4 and p70S6K Signaling | 2.512×10^-11^ | 0.447 |
| Coronavirus Pathogenesis Pathway | 2.455×10^-07^ | 4.013 |
| mTOR Signaling | 9.120×10^-07^ | 0 |
| B Cell Receptor Signaling | 9.120×10^-07^ | 2.492 |
| Th1 and Th2 Activation Pathway | 1.479×10^-06^ | NA |
| Natural Killer Cell Signaling | 2.630×10^-06^ | 0.671 |
| Th1 Pathway | 5.623×10^-05^ | -0.152 |
| Th2 Pathway | 5.623×10^-05^ | 1.809 |
| STAT3 Pathway | 3.802×10^-04^ | 1.508 |
| HGF Signaling | 8.128×10^-04^ | 1.581 |
| Role of PKR in Interferon Induction and Antiviral Response | 9.120×10^-04^ | 2.1 |
| HIF1α Signaling | 9.772×10^-04^ | 2.79 |
| PPAR Signaling | 1.413×10^-03^ | -0.457 |
| RANK Signaling in Osteoclasts | 2.630×10^-03^ | 2.058 |

**Supplementary Table 7** Genes with additive behaviour in addition to being significant in all pairwise comparisons whilst correcting for immunomodulatory treatment. Direction of ‘up’ means their levels increase with increasing severity.

| **ID** | **Gene** | **Direction** |
| --- | --- | --- |
| ENSG00000204933 | CD177P1 | Up |
| ENSG00000169174 | PCSK9 | Up |
| ENSG00000102837 | OLFM4 | Up |
| ENSG00000204936 | CD177 | Up |
| ENSG00000118113 | MMP8 | Up |
| ENSG00000163710 | PCOLCE2 | Up |
| ENSG00000179869 | ABCA13 | Up |
| ENSG00000065618 | COL17A1 | Up |
| ENSG00000170439 | METTL7B | Up |
| ENSG00000242550 | SERPINB10 | Up |
| ENSG00000064270 | ATP2C2 | Up |
| ENSG00000268833 | - | Up |
| ENSG00000211965 | IGHV3-49 | Up |
| ENSG00000069535 | MAOB | Up |
| ENSG00000211665 | IGLV3-16 | Up |
| ENSG00000211937 | IGHV2-5 | Up |
| ENSG00000160180 | TFF3 | Up |
| ENSG00000224940 | PRRT4 | Up |
| ENSG00000241244 | IGKV1D-16 | Up |
| ENSG00000232216 | IGHV3-43 | Up |
| ENSG00000136010 | ALDH1L2 | Up |
| ENSG00000211959 | IGHV4-39 | Up |
| ENSG00000139572 | GPR84 | Up |
| ENSG00000278196 | IGLV2-8 | Up |
| ENSG00000224650 | IGHV3-74 | Up |
| ENSG00000104918 | RETN | Up |
| ENSG00000279400 | - | Up |
| ENSG00000182175 | RGMA | Up |
| ENSG00000106853 | PTGR1 | Up |
| ENSG00000133063 | CHIT1 | Up |
| ENSG00000158352 | SHROOM4 | Up |
| ENSG00000005961 | ITGA2B | Up |
| ENSG00000079393 | DUSP13 | Up |
| ENSG00000275898 | - | Up |
| ENSG00000164850 | GPER1 | Up |
| ENSG00000250280 | - | Up |
| ENSG00000163661 | PTX3 | Up |
| ENSG00000265688 | MAFG-DT | Up |
| ENSG00000168528 | SERINC2 | Up |
| ENSG00000122861 | PLAU | Up |
| ENSG00000162407 | PLPP3 | Up |
| ENSG00000070371 | CLTCL1 | Up |
| ENSG00000109265 | CRACD | Up |
| ENSG00000137563 | GGH | Up |
| ENSG00000228923 | - | Up |
| ENSG00000197653 | DNAH10 | Up |
| ENSG00000250644 | - | Up |
| ENSG00000100024 | UPB1 | Up |
| ENSG00000135862 | LAMC1 | Up |
| ENSG00000223916 | - | Up |
| ENSG00000105711 | SCN1B | Up |
| ENSG00000111186 | WNT5B | Up |
| ENSG00000144407 | PTH2R | Up |
| ENSG00000184702 | SEPTIN5 | Up |
| ENSG00000226928 | RPS14P4 | Up |
| ENSG00000279276 | - | Up |
| ENSG00000051128 | HOMER3 | Up |
| ENSG00000152380 | FAM151B | Up |
| ENSG00000185909 | KLHDC8B | Up |
| ENSG00000197063 | MAFG | Up |
| ENSG00000280167 | - | Up |
| ENSG00000008394 | MGST1 | Up |
| ENSG00000111181 | SLC6A12 | Up |
| ENSG00000072954 | TMEM38A | Up |
| ENSG00000104043 | ATP8B4 | Up |
| ENSG00000228817 | BACH1-IT2 | Up |
| ENSG00000229512 | - | Up |
| ENSG00000064601 | CTSA | Up |
| ENSG00000075651 | PLD1 | Up |
| ENSG00000258337 | - | Up |
| ENSG00000118985 | ELL2 | Up |
| ENSG00000271795 | - | Up |
| ENSG00000108405 | P2RX1 | Up |
| ENSG00000164236 | ANKRD33B | Up |
| ENSG00000221598 | MIR1249 | Up |
| ENSG00000248476 | BACH1-IT1 | Up |
| ENSG00000199805 | RNU1-134P | Up |
| ENSG00000263120 | - | Up |
| ENSG00000262580 | - | Up |
| ENSG00000277255 | MIR7854 | Up |
| ENSG00000227200 | - | Up |
| ENSG00000067225 | PKM | Up |
| ENSG00000127838 | PNKD | Up |
| ENSG00000279476 | - | Up |
| ENSG00000181523 | SGSH | Up |
| ENSG00000243508 | DNAJB6P7 | Up |
| ENSG00000198018 | ENTPD7 | Up |
| ENSG00000267632 | - | Up |
| ENSG00000070214 | SLC44A1 | Up |
| ENSG00000162341 | TPCN2 | Up |
| ENSG00000166340 | TPP1 | Up |
| ENSG00000198113 | TOR4A | Up |
| ENSG00000278949 | - | Up |
| ENSG00000165714 | BORCS5 | Up |
| ENSG00000196295 | GARS1-DT | Up |
| ENSG00000164961 | WASHC5 | Up |
| ENSG00000154001 | PPP2R5E | Down |
| ENSG00000253719 | ATXN7L3B | Down |
| ENSG00000183513 | COA5 | Down |
| ENSG00000172262 | ZNF131 | Down |
| ENSG00000165943 | MOAP1 | Down |
| ENSG00000139163 | ETNK1 | Down |
| ENSG00000149311 | ATM | Down |
| ENSG00000107771 | CCSER2 | Down |
| ENSG00000149313 | AASDHPPT | Down |
| ENSG00000198252 | STYX | Down |
| ENSG00000198791 | CNOT7 | Down |
| ENSG00000186104 | CYP2R1 | Down |
| ENSG00000047932 | GOPC | Down |
| ENSG00000173726 | TOMM20 | Down |
| ENSG00000104979 | C19orf53 | Down |
| ENSG00000119335 | SET | Down |
| ENSG00000066654 | THUMPD1 | Down |
| ENSG00000204977 | TRIM13 | Down |
| ENSG00000110696 | C11orf58 | Down |
| ENSG00000138767 | CNOT6L | Down |
| ENSG00000150593 | PDCD4 | Down |
| ENSG00000139154 | AEBP2 | Down |
| ENSG00000144034 | TPRKB | Down |
| ENSG00000122034 | GTF3A | Down |
| ENSG00000083535 | PIBF1 | Down |
| ENSG00000080822 | CLDND1 | Down |
| ENSG00000116750 | UCHL5 | Down |
| ENSG00000129317 | PUS7L | Down |
| ENSG00000163322 | ABRAXAS1 | Down |
| ENSG00000126804 | ZBTB1 | Down |
| ENSG00000115419 | GLS | Down |
| ENSG00000147679 | UTP23 | Down |
| ENSG00000148362 | PAXX | Down |
| ENSG00000107625 | DDX50 | Down |
| ENSG00000189227 | C15orf61 | Down |
| ENSG00000198331 | HYLS1 | Down |
| ENSG00000137770 | CTDSPL2 | Down |
| ENSG00000165156 | ZHX1 | Down |
| ENSG00000122484 | RPAP2 | Down |
| ENSG00000163607 | GTPBP8 | Down |
| ENSG00000171490 | RSL1D1 | Down |
| ENSG00000167842 | MIS12 | Down |
| ENSG00000170903 | MSANTD4 | Down |
| ENSG00000170364 | SETMAR | Down |
| ENSG00000214367 | HAUS3 | Down |
| ENSG00000197894 | ADH5 | Down |
| ENSG00000166037 | CEP57 | Down |
| ENSG00000161016 | RPL8 | Down |
| ENSG00000251022 | THAP9-AS1 | Down |
| ENSG00000122873 | CISD1 | Down |
| ENSG00000144895 | EIF2A | Down |
| ENSG00000106460 | TMEM106B | Down |
| ENSG00000204387 | SNHG32 | Down |
| ENSG00000162244 | RPL29 | Down |
| ENSG00000005469 | CROT | Down |
| ENSG00000255559 | ZNF252P-AS1 | Down |
| ENSG00000162623 | TYW3 | Down |
| ENSG00000117906 | RCN2 | Down |
| ENSG00000111875 | ASF1A | Down |
| ENSG00000188846 | RPL14 | Down |
| ENSG00000151332 | MBIP | Down |
| ENSG00000197056 | ZMYM1 | Down |
| ENSG00000105193 | RPS16 | Down |
| ENSG00000166226 | CCT2 | Down |
| ENSG00000182141 | ZNF708 | Down |
| ENSG00000226287 | TMEM191A | Down |
| ENSG00000273015 | - | Down |
| ENSG00000074935 | TUBE1 | Down |
| ENSG00000164022 | AIMP1 | Down |
| ENSG00000254838 | GVINP1 | Down |
| ENSG00000256087 | ZNF432 | Down |
| ENSG00000139324 | TMTC3 | Down |
| ENSG00000100442 | FKBP3 | Down |
| ENSG00000105829 | BET1 | Down |
| ENSG00000090612 | ZNF268 | Down |
| ENSG00000088179 | PTPN4 | Down |
| ENSG00000139343 | SNRPF | Down |
| ENSG00000133641 | C12orf29 | Down |
| ENSG00000138660 | AP1AR | Down |
| ENSG00000156017 | CARNMT1 | Down |
| ENSG00000213186 | TRIM59 | Down |
| ENSG00000109971 | HSPA8 | Down |
| ENSG00000180257 | ZNF816 | Down |
| ENSG00000180917 | CMTR2 | Down |
| ENSG00000120694 | HSPH1 | Down |
| ENSG00000245910 | SNHG6 | Down |
| ENSG00000110700 | RPS13 | Down |
| ENSG00000115816 | CEBPZ | Down |
| ENSG00000182287 | AP1S2 | Down |
| ENSG00000090266 | NDUFB2 | Down |
| ENSG00000174748 | RPL15 | Down |
| ENSG00000114686 | MRPL3 | Down |
| ENSG00000166275 | BORCS7 | Down |
| ENSG00000149273 | RPS3 | Down |
| ENSG00000083845 | RPS5 | Down |
| ENSG00000120686 | UFM1 | Down |
| ENSG00000197958 | RPL12 | Down |
| ENSG00000134049 | IER3IP1 | Down |
| ENSG00000170846 | - | Down |
| ENSG00000164172 | MOCS2 | Down |
| ENSG00000221944 | TIGD1 | Down |
| ENSG00000177721 | ANXA2R | Down |
| ENSG00000058729 | RIOK2 | Down |
| ENSG00000233757 | - | Down |
| ENSG00000182359 | KBTBD3 | Down |
| ENSG00000018869 | ZNF582 | Down |
| ENSG00000147403 | RPL10 | Down |
| ENSG00000105849 | POLR1F | Down |
| ENSG00000144713 | RPL32 | Down |
| ENSG00000158691 | ZSCAN12 | Down |
| ENSG00000075089 | ACTR6 | Down |
| ENSG00000167232 | ZNF91 | Down |
| ENSG00000198440 | ZNF583 | Down |
| ENSG00000180787 | ZFP3 | Down |
| ENSG00000152133 | GPATCH11 | Down |
| ENSG00000106591 | MRPL32 | Down |
| ENSG00000123545 | NDUFAF4 | Down |
| ENSG00000241127 | YAE1 | Down |
| ENSG00000163577 | EIF5A2 | Down |
| ENSG00000104408 | EIF3E | Down |
| ENSG00000113966 | ARL6 | Down |
| ENSG00000197050 | ZNF420 | Down |
| ENSG00000259834 | - | Down |
| ENSG00000168283 | BMI1 | Down |
| ENSG00000083099 | LYRM2 | Down |
| ENSG00000254004 | ZNF260 | Down |
| ENSG00000255135 | - | Down |
| ENSG00000198707 | CEP290 | Down |
| ENSG00000177888 | ZBTB41 | Down |
| ENSG00000168116 | KIAA1586 | Down |
| ENSG00000151835 | SACS | Down |
| ENSG00000137038 | DMAC1 | Down |
| ENSG00000172172 | MRPL13 | Down |
| ENSG00000198346 | ZNF813 | Down |
| ENSG00000168028 | RPSA | Down |
| ENSG00000143971 | ETAA1 | Down |
| ENSG00000169288 | MRPL1 | Down |
| ENSG00000174444 | RPL4 | Down |
| ENSG00000163281 | GNPDA2 | Down |
| ENSG00000179144 | GIMAP7 | Down |
| ENSG00000101132 | PFDN4 | Down |
| ENSG00000186468 | RPS23 | Down |
| ENSG00000142937 | RPS8 | Down |
| ENSG00000100316 | RPL3 | Down |
| ENSG00000137501 | SYTL2 | Down |
| ENSG00000120526 | NUDCD1 | Down |
| ENSG00000140006 | WDR89 | Down |
| ENSG00000116791 | CRYZ | Down |
| ENSG00000142541 | RPL13A | Down |
| ENSG00000198464 | ZNF480 | Down |
| ENSG00000177932 | ZNF354C | Down |
| ENSG00000182774 | RPS17 | Down |
| ENSG00000122406 | RPL5 | Down |
| ENSG00000136897 | MRPL50 | Down |
| ENSG00000196911 | KPNA5 | Down |
| ENSG00000261366 | MANEA-DT | Down |
| ENSG00000236552 | RPL13AP5 | Down |
| ENSG00000164587 | RPS14 | Down |
| ENSG00000152219 | ARL14EP | Down |
| ENSG00000197841 | ZNF181 | Down |
| ENSG00000118181 | RPS25 | Down |
| ENSG00000067840 | PDZD4 | Down |
| ENSG00000167286 | CD3D | Down |
| ENSG00000163519 | TRAT1 | Down |
| ENSG00000156508 | EEF1A1 | Down |
| ENSG00000111678 | C12orf57 | Down |
| ENSG00000174946 | GPR171 | Down |
| ENSG00000142875 | PRKACB | Down |
| ENSG00000078596 | ITM2A | Down |
| ENSG00000143947 | RPS27A | Down |
| ENSG00000169740 | ZNF32 | Down |
| ENSG00000008988 | RPS20 | Down |
| ENSG00000244720 | NT5C3AP2 | Down |
| ENSG00000146757 | ZNF92 | Down |
| ENSG00000164114 | MAP9 | Down |
| ENSG00000213741 | RPS29 | Down |
| ENSG00000137154 | RPS6 | Down |
| ENSG00000196205 | EEF1A1P5 | Down |
| ENSG00000007264 | MATK | Down |
| ENSG00000212802 | RPL15P3 | Down |
| ENSG00000214194 | SMIM30 | Down |
| ENSG00000165169 | DYNLT3 | Down |
| ENSG00000127184 | COX7C | Down |
| ENSG00000182899 | RPL35A | Down |
| ENSG00000136942 | RPL35 | Down |
| ENSG00000125245 | GPR18 | Down |
| ENSG00000270638 | - | Down |
| ENSG00000233476 | EEF1A1P6 | Down |
| ENSG00000269893 | SNHG8 | Down |
| ENSG00000165512 | ZNF22 | Down |
| ENSG00000150045 | KLRF1 | Down |
| ENSG00000156482 | RPL30 | Down |
| ENSG00000169508 | GPR183 | Down |
| ENSG00000231500 | RPS18 | Down |
| ENSG00000198574 | SH2D1B | Down |
| ENSG00000183918 | SH2D1A | Down |
| ENSG00000169442 | CD52 | Down |
| ENSG00000171858 | RPS21 | Down |
| ENSG00000179841 | AKAP5 | Down |
| ENSG00000198756 | COLGALT2 | Down |
| ENSG00000110848 | CD69 | Down |
| ENSG00000224631 | RPS27AP16 | Down |
| ENSG00000113088 | GZMK | Down |
| ENSG00000139679 | LPAR6 | Down |
| ENSG00000242299 | - | Down |
| ENSG00000177954 | RPS27 | Down |
| ENSG00000162620 | LRRIQ3 | Down |
| ENSG00000145649 | GZMA | Down |
| ENSG00000279377 | - | Down |
| ENSG00000111796 | KLRB1 | Down |
| ENSG00000147604 | RPL7 | Down |

**Supplementary Table 8** The genes identified as SDE with age with severity and the interaction between age and severity included in the model. Positive and negative log2 fold change indicate increasing and decreasing levels with increasing age.

| **ID** | **Gene** | **log2FoldChange** | **B-H p-value** |
| --- | --- | --- | --- |
| ENSG00000242076 | IGKV1-33 | 0.1549 | 2.126E-02 |
| ENSG00000204001 | LCN8 | 0.1706 | 2.126E-02 |
| ENSG00000048052 | HDAC9 | 0.0323 | 2.280E-02 |
| ENSG00000135116 | HRK | 0.1177 | 2.280E-02 |
| ENSG00000272871 | NA | 0.0494 | 2.424E-02 |
| ENSG00000228526 | MIR34AHG | 0.0677 | 2.804E-02 |
| ENSG00000170390 | DCLK2 | 0.1229 | 2.804E-02 |
| ENSG00000124429 | POF1B | 0.0948 | 2.804E-02 |
| ENSG00000102595 | UGGT2 | 0.0410 | 2.804E-02 |
| ENSG00000154262 | ABCA6 | 0.1337 | 2.804E-02 |
| ENSG00000079337 | RAPGEF3 | 0.0893 | 2.927E-02 |
| ENSG00000172869 | DMXL1 | 0.0136 | 3.181E-02 |
| ENSG00000169385 | RNASE2 | 0.0594 | 3.181E-02 |
| ENSG00000100077 | GRK3 | 0.0230 | 3.181E-02 |
| ENSG00000165507 | DEPP1 | 0.1496 | 3.193E-02 |
| ENSG00000104472 | CHRAC1 | -0.0131 | 3.232E-02 |
| ENSG00000196440 | ARMCX4 | 0.0197 | 3.304E-02 |
| ENSG00000156869 | FRRS1 | 0.0308 | 3.993E-02 |
| ENSG00000118432 | CNR1 | 0.1063 | 4.097E-02 |
| ENSG00000198947 | DMD | 0.1304 | 4.097E-02 |
| ENSG00000108950 | FAM20A | 0.1076 | 4.097E-02 |
| ENSG00000210082 | MT-RNR2 | -0.1011 | 4.098E-02 |
| ENSG00000064652 | SNX24 | 0.0243 | 4.388E-02 |
| ENSG00000185710 | SMG1P4 | -0.0801 | 4.388E-02 |
| ENSG00000154258 | ABCA9 | 0.1245 | 4.388E-02 |

**Full list of the PERFORM Consortium**

**PARTNER: IMPERIAL COLLEGE (UK)**

Chief investigator/PERFORM coordinator:

Michael Levin

Principal and co-investigators; work package leads (alphabetical order)

Aubrey Cunnington (grant application)

Tisham De (work package lead)

Jethro Herberg (Principle Investigator, Deputy Coordinator, grant application)

Myrsini Kaforou (grant application, work package lead)

Victoria Wright (grant application, Scientific Coordinator)

Research Group (alphabetical order)

Lucas Baumard; Evangelos Bellos; Giselle D’Souza; Rachel Galassini; Dominic Habgood-Coote; Shea Hamilton; Clive Hoggart; Sara Hourmat; Heather Jackson; Ian Maconochie; Stephanie Menikou; Naomi Lin; Samuel Nichols; Ruud Nijman; Ivonne Pena Paz; Priyen Shah; Ching-Fen Shen; Clare Wilson

Clinical recruitment at Imperial College Healthcare NHS Trust (alphabetical order)

Amina Abdulla; Ladan Ali; Sarah Darnell; Rikke Jorgensen; Sobia Mustafa; Salina Persand

Imperial College Faculty of Engineering

Molly Stevens (co-investigator), Eunjung Kim (research group); Benjamin Pierce (research group)

Clinical recruitment at Brighton and Sussex University Hospitals

Katy Fidler (Principle Investigator)

Julia Dudley (Clinical Research Registrar)

Research nurses: Vivien Richmond, Emma Tavliavini

Clinical recruitment at National Cheng Kung University Hospital

Ching-Fen Shen (Principal Investigator); Ching-Chuan Liu (Co-investigator); Shih-Min Wang (Co-investigator), funded by the Center of Clinical Medicine Research, National Cheng Kung University

**PARTNER: SERGAS (Spain)**

Principal Investigators

Federico Martinón-Torres^1^

Antonio Salas^1,2^

Research Group (alphabetical order)

Fernando Álvez González^1^, Cristina Balo Farto^1^, Ruth Barral-Arca^1,2^, María Barreiro Castro^1^, Xabier Bello^1,2^, Mirian Ben García^1^, Sandra Carnota^1^, Miriam Cebey-López^1^, María José Curras-Tuala^1,2^, Carlos Durán Suárez^1^, Luisa García Vicente^1^, Alberto Gómez-Carballa^1,2^, Jose Gómez Rial^1^, Pilar Leboráns Iglesias^1^, Federico Martinón-Torres^1^, Nazareth Martinón-Torres^1^, José María Martinón Sánchez^1^, Belén Mosquera Pérez^1^, Jacobo Pardo-Seco^1,2^, Lidia Piñeiro Rodríguez^1^, Sara Pischedda^1,2^, Sara Rey Vázquez^1^, Irene Rivero Calle^1^, Carmen Rodríguez-Tenreiro^1^, Lorenzo Redondo-Collazo^1^, Miguel Sadiki Ora^1^, Antonio Salas^1,2^, Sonia Serén Fernández^1^, Cristina Serén Trasorras^1^, Marisol Vilas Iglesias^1^.

^1^ Translational Pediatrics and Infectious Diseases, Pediatrics Department, Hospital Clínico Universitario de Santiago, Santiago de Compostela, Spain, and GENVIP Research Group (www.genvip.org), Instituto de Investigación Sanitaria de Santiago, Universidad de Santiago de Compostela, Galicia, Spain.

^2^ Unidade de Xenética, Departamento de Anatomía Patolóxica e Ciencias Forenses, Instituto de Ciencias Forenses, Facultade de Medicina, Universidade de Santiago de Compostela, and GenPop Research Group, Instituto de Investigaciones Sanitarias (IDIS), Hospital Clínico Universitario de Santiago, Galicia, Spain

^3^ Fundación Pública Galega de Medicina Xenómica, Servizo Galego de Saúde (SERGAS), Instituto de Investigaciones Sanitarias (IDIS), and Grupo de Medicina Xenómica, Centro de Investigación Biomédica en Red de Enfermedades Raras (CIBERER), Universidade de Santiago de Compostela (USC), Santiago de Compostela, Spain

**PARTNER: RSU (Latvia)**

Principal Investigator

Dace Zavadska^1,2^

Other RSU group authors (in alphabetical order):

Anda Balode^1,2^, Arta Bārzdiņa^1,2^, Dārta Deksne^1,2^, Dace Gardovska^1,2^, Dagne Grāvele^2^, Ilze Grope^1,2^, Anija Meiere^1,2^, Ieva Nokalna^1,2^, Jana Pavāre^1,2^, Zanda Pučuka^1,2^, Katrīna Selecka^1,2^, Aleksandra Sidorova^1,2^, Dace Svile^2^, Urzula Nora Urbāne^1,2^.

^1^ Riga Stradins university, Riga, Latvia.

^2^ Children clinical university hospital, Riga, Latvia.

**PARTNER: Medical Research Council Unit The Gambia (MRCG) at LSHTM**

Principal Investigator

Effua Usuf

Additional Investigators

Kalifa Bojang

Syed M. A. Zaman

Fatou Secka

Suzanne Anderson

Anna RocaIsatou Sarr

Momodou Saidykhan

Saffiatou Darboe

Samba Ceesay

Umberto D’alessandro

Medical Research Council Unit The Gambia at LSHTM

P O Box 273,

Fajara, The Gambia

**PARTNER: ERASMUS MC-Sophia Children’s Hospital (Netherlands**

Principal Investigator

Henriëtte A. Moll¹

Research Group (alphabetical order)

Dorine M. Borensztajn¹, Nienke N. Hagedoorn, Chantal Tan ¹, ¹, Clementien L. Vermont², Joany Zachariasse ¹

Additional investigator

W Dik ^3^

¹ Erasmus MC-Sophia Children’s Hospital, Department of General Paediatrics, Rotterdam, the Netherlands

² Erasmus MC-Sophia Children’s Hospital, Department of Paediatric Infectious Diseases & Immunology, Rotterdam, the Netherlands

^3^ Erasmus MC, Department of immunology, Rotterdam, the Netherlands

**PARTNER: Swiss Pediatric Sepsis Study (Switzerland)**

Principal Investigators*:*

Philipp Agyeman, MD ^1^ (ORCID 0000-0002-8339-5444), Luregn J Schlapbach, MD, FCICM ^2,3^ (ORCID 0000-0003-2281-2598)

Clinical recruitment at University Children’s Hospital Bern for PERFORM:

Christoph Aebi ^1^, Verena Wyss ^1^, Mariama Usman ^1^

Principal and co-investigators for the Swiss Pediatric Sepsis Study:

Philipp Agyeman, MD ^1^, Luregn J Schlapbach, MD, FCICM ^2,3^, Eric Giannoni, MD ^4,5^, Martin Stocker, MD ^6^, Klara M Posfay-Barbe, MD ^7^, Ulrich Heininger, MD ^8^, Sara Bernhard-Stirnemann, MD ^9^, Anita Niederer-Loher, MD ^10^, Christian Kahlert, MD ^10^, Giancarlo Natalucci, MD ^11^, Christa Relly, MD ^12^, Thomas Riedel, MD ^13^, Christoph Aebi, MD ^1^, Christoph Berger, MD ^12^ for the Swiss Pediatric Sepsis Study

^1^ Department of Pediatrics, Inselspital, Bern University Hospital, University of Bern, Switzerland

^2^ Neonatal and Pediatric Intensive Care Unit, Children’s Research Center, University Children’s Hospital Zurich, University of Zurich, Zurich, Switzerland

^3^Child Health Research Centre, University of Queensland, and Queensland Children`s Hospital, Brisbane, Australia

^4^ Clinic of Neonatology, Department Mother-Woman-Child, Lausanne University Hospital and University of Lausanne, Switzerland

^5^ Infectious Diseases Service, Department of Medicine, Lausanne University Hospital and University of Lausanne, Switzerland

^6^ Department of Pediatrics, Children’s Hospital Lucerne, Lucerne, Switzerland

^7^ Pediatric Infectious Diseases Unit, Children’s Hospital of Geneva, University Hospitals of Geneva, Geneva, Switzerland

^8^ Infectious Diseases and Vaccinology, University of Basel Children’s Hospital, Basel, Switzerland

^9^ Children’s Hospital Aarau, Aarau, Switzerland

^10^ Division of Infectious Diseases and Hospital Epidemiology, Children’s Hospital of Eastern Switzerland St. Gallen, St. Gallen, Switzerland

^11^ Department of Neonatology, University Hospital Zurich, Zurich, Switzerland

^12^ Division of Infectious Diseases and Hospital Epidemiology, and Children’s Research Center, University Children’s Hospital Zurich, Switzerland

^13^ Children’s Hospital Chur, Chur, Switzerland

**PARTNER: Liverpool (UK)**

Principal Investigators

Enitan D Carrol^1,2,3^

Stéphane Paulus ^1,^

Research Group (alphabetical order)

Elizabeth Cocklin^1^, Rebecca Jennings^4^, Joanne Johnston^4^, Simon Leigh^1^, Karen Newall^4^, Sam Romaine^1^

^1^ Department of Clinical Infection, Microbiology and Immunology, University of Liverpool Institute of Infection and Global Health , Liverpool, England

^2^ Alder Hey Children’s Hospital, Department of Infectious Diseases, Eaton Road, Liverpool, L12 2AP

^3^ Liverpool Health Partners, 1st Floor, Liverpool Science Park, 131 Mount Pleasant, Liverpool, L3 5TF

^4^Alder Hey Children’s Hospital, Clinical Research Business Unit, Eaton Road, Liverpool, L12 2AP

**PARTNER: NKUA (Greece)**

Principal investigator

Professor Maria Tsolia (all activities)

Investigator/Research fellow

Irini Eleftheriou (all activities)

Additional investigators

Recruitment: Maria Tambouratzi

Lab: Antonis Marmarinos (Quality Manager)

Lab: Marietta Xagorari

Kelly Syggelou

2nd Department of Pediatrics, National and Kapodistrian University of Athens,

“P. and A. Kyriakou” Children’s Hospital

Thivon and Levadias

Goudi, Athens

**PARTNER: Micropathology Ltd (UK)**

Principal Investigator

Professor Colin Fink^1^, Clinical Microbiologist

Additional investigators

Dr Marie Voice^1^, Post doc scientist

Dr. Leo Calvo-Bado^1^, Post doc scientist

^1^ Micropathology Ltd, The Venture Center, University of Warwick Science Park, Sir William Lyons Road, Coventry, CV4 7EZ.

**PARTNER: Medical University of Graz (MUG, Austria)**

Principal Investigator

Werner Zenz^1^ (all activities)

Co-investigators (alphabetical order)

Benno Kohlmaier^1^ (all activities)

Nina A. Schweintzger^1^ (all activities)

Manfred G. Sagmeister^1^ (study design, consortium wide sample management)

Research team

Daniela S. Kohlfürst^1^ (study design)

Christoph Zurl^1^ (BIVA PIC)

Alexander Binder^1^ (grant application)

Recruitment team, data managers, (alphabetical order)

Susanne Hösele^1^, Manuel Leitner^1^, Lena Pölz^1^, Glorija Rajic^1^,

Clinical recruitment partners (alphabetical order)

Sebastian Bauchinger^1^, Hinrich Baumgart^4^, Martin Benesch^3^, Astrid Ceolotto^1^, Ernst Eber^2^, Siegfried Gallistl^1^, Gunther Gores^5^, Harald Haidl^1^, Almuthe Hauer^1^, Christa Hude^1^, Markus Keldorfer^5^, Larissa Krenn^4^, Heidemarie Pilch^5^, Andreas Pfleger^2^, Klaus Pfurtscheller^4^, Gudrun Nordberg^5^, Tobias Niedrist^8^, Siegfried Rödl^4^, Andrea Skrabl-Baumgartner^1^, Matthias Sperl^7^, Laura Stampfer^5^, Volker Strenger^3^, Holger Till^6^, Andreas Trobisch^5^, Sabine Löffler^5^

^1^ Department of Pediatrics and Adolescent Medicine, Division of General Pediatrics, Medical University of Graz, Graz, Austria

^2^Department of Pediatric Pulmonology, Medical University of Graz, Graz, Austria

^3^Department of Pediatric Hematooncoloy, Medical University of Graz, Graz, Austria

^4^Paediatric Intensive Care Unit, Medical University of Graz, Graz, Austria

^5^University Clinic of Paediatrics and Adolescent Medicine Graz, Medical University Graz, Graz,Austria

^6^Department of Paediatric and Adolescence Surgery, Medical University Graz, Graz, Austria

^7^Department of Pediatric Orthopedics, Medical University Graz, Graz, Austria

^8^Clinical Institute of Medical and Chemical Laboratory Diagnostics, Medical University Graz, Graz, Austria

**PARTNER: London School of Hygiene and Tropical Medicine (UK)**

WP 1 WP2, WP5

Principal Investigator:

Dr Shunmay Yeung^1,2 3^ PhD, MBBS, FRCPCH, MRCP, DTM&H

Research Group

Dr Juan Emmanuel Dewez^1^ MD, DTM&H, MSc

Prof Martin Hibberd ^1^ BSc, PhD

Mr David Bath^2^ MSc, MAppFin, BA(Hons)

Dr Alec Miners^2^ BA(Hons), MSc, PhD

Dr Ruud Nijman^3^ PhD MSc MD MRCPCH

Dr Catherine Wedderburn^1^ BA, MBChB, DTM&H, MSc, MRCPCH

Ms Anne Meierford^1^ MSc, BMedSc, BMBS

Dr Baptiste Leurent^4^, PhD, MSc

1. Faculty of Infectious and Tropical Disease, London School of Hygiene and Tropical Medicine, London, UK
2. Faculty of Public Health and Policy, London School of Hygiene and Tropical Medicine, London, UK
3. Department of Paediatrics, St. Mary’s Hospital Imperial College Hospital, London, UK
4. Faculty of Epidemiology and Population Health, London School of Hygiene and Tropical Medicine, London, UK

**PARTNER: Radboud University Medical Center (RUMC, Netherlands)**

Principal Investigators

Ronald de Groot ^1^, Michiel van der Flier ^1,2,3^, Marien I. de Jonge^1^

Co-investigators Radboud University Medical Center (alphabetical order)

Koen van Aerde^1,2^, Wynand Alkema^1^, Bryan van den Broek^1^, Jolein Gloerich^1^, Alain J. van Gool^1^, Stefanie Henriet^1,2^, Martijn Huijnen^1^, Ria Philipsen^1^, Esther Willems^1^

Investigators PeDBIG PERFORM DUTCH CLINICAL NETWORK (alphabetical order)

G.P.J.M. Gerrits^8^, M. van Leur^8,^ J. Heidema ^4^,L. de Haan^1,2^ C.J. Miedema ^5^, C. Neeleman ^1^ C.C. Obihara ^6^, G.A. Tramper-Stranders7^6^

1. Radboud University Medical Center, Nijmegen, The Netherlands
2. Amalia Children’s Hospital, Nijmegen, The Netherlands
3. Wilhelmina Children’s Hospital, University Medical Center Utrecht, Utrecht, The Netherlands
4. St. Antonius Hospital, Nieuwegein, The Netherlands
5. Catharina Hospital, Eindhoven, The Netherlands
6. ETZ Elisabeth, Tilburg, The Netherlands
7. Franciscus Gasthuis, Rotterdam, The Netherlands
8. Canisius Wilhelmina Hospital, Nijmegen, The Netherlands

**PARTNER: Oxford (UK)**

Principal Investigators

Andrew J. Pollard^1,2^, Rama Kandasamy^1,2^, Stéphane Paulus ^1,2^

Additional Investigators

Michael J. Carter^1,2^, Daniel O'Connor^1,2^, Sagida Bibi^1,2^, Dominic F. Kelly^1,2^, Meeru Gurung^3^, Stephen Thorson^3^, Imran Ansari^3^, David R. Murdoch^4^, Shrijana Shrestha^3^.

^1^Oxford Vaccine Group, Department of Paediatrics, University of Oxford, Oxford, United Kingdom.

^2^NIHR Oxford Biomedical Research Centre, Oxford, United Kingdom.

^3^Paediatric Research Unit, Patan Academy of Health Sciences, Kathmandu, Nepal.

^4^Department of Pathology, University of Otago, Christchurch, New Zealand.

**PARTNER: Newcastle University, Newcastle upon Tyne, (UK)**

Principal Investigator

Marieke Emonts ^1,2,3^ (all activities)

Co-investigators

Emma Lim^2,3,7^ (all activities)

Lucille Valentine^4^

Recruitment team (alphabetical), data-managers, and GNCH Research unit

Karen Allen^5^, Kathryn Bell^5^, Adora Chan^5^, Stephen Crulley^5^, Kirsty Devine^5^, Daniel Fabian^5^, Sharon King^5^, Paul McAlinden^5^, Sam McDonald^5^, Anne McDonnell2,^5^, Ailsa Pickering^2,5^, Evelyn Thomson^5^, Amanda Wood^5^, Diane Wallia^5^, Phil Woodsford^5^,

Sample processing: Frances Baxter^5^, Ashley Bell^5^, Mathew Rhodes^5^

PICU recruitment

Rachel Agbeko^8^

Christine Mackerness^8^

Students MOFICHE

Bryan Baas^2^, Lieke Kloosterhuis^2^, Wilma Oosthoek^2^

Students/medical staff PERFORM

Tasnim Arif^6^, Joshua Bennet^2^, Kalvin Collings^2^, Ilona van der Giessen^2^, Alex Martin^2^, Aqeela Rashid^6^, Emily Rowlands^2^, Gabriella de Vries^2^, Fabian van der Velden^2^

Engagement work/ethics/cost effectiveness

Lucille Valentine ^4^, Mike Martin^9^, Ravi Mistry^2^, Lucille Valentine^4^

^1^ Translational and Clinical Research Institute, Newcastle University, Newcastle upon Tyne UK

^2^Great North Children’s Hospital, Paediatric Immunology, Infectious Diseases & Allergy, Newcastle upon Tyne Hospitals NHS Foundation Trust, Newcastle upon Tyne, United Kingdom.

^3^NIHR Newcastle Biomedical Research Centre based at Newcastle upon Tyne Hospitals NHS Trust and Newcastle University, Westgate Rd, Newcastle upon Tyne NE4 5PL, United Kingdom

^4^Newcastle University Business School, Centre for Knowledge, Innovation, Technology and Enterprise (KITE), Newcastle upon Tyne, United Kingdom

^5^Great North Children’s Hospital, Research Unit, Newcastle upon Tyne Hospitals NHS Foundation Trust, Newcastle upon Tyne, United Kingdom.

^6^Great North Children’s Hospital, Paediatric Oncology, Newcastle upon Tyne Hospitals NHS Foundation Trust, Newcastle upon Tyne, United Kingdom.

^7^Population Health Sciences Institute, Newcastle University, Newcastle upon Tyne, UK

^8^Great North Children’s Hospital, Paediatric Intensive Care Unit, Newcastle upon Tyne Hospitals NHS Foundation Trust, Newcastle upon Tyne, United Kingdom.

^9^Northumbria University, Newcastle upon Tyne, United Kingdom.

**PARTNER: LMU Munich (Germany)**

Principal Investigator

Ulrich von Both^1,2^ MD, FRCPCH (all activities)

Research group

Laura Kolberg¹ MSc (all activities)

Manuela Zwerenz¹ MSc, Judith Buschbeck¹ PhD

Clinical recruitment partners (alphabetical order)

Christoph Bidlingmaier^3^, Vera Binder^4^, Katharina Danhauser^5^, Nikolaus Haas^10^, Matthias Griese^6^, Tobias Feuchtinger^4^, Julia Keil^9^, Matthias Kappler^6^, Eberhard Lurz^7^, Georg Muench^8^, Karl Reiter^9^, Carola Schoen^9^

¹Div. Paediatric Infectious Diseases, Hauner Children’s Hospital, University Hospital, Ludwig Maximilians University (LMU), Munich, Germany

^2^German Center for Infection Research (DZIF), Partner Site Munich, Munich, Germany

^3^Div. of General Paediatrics, ^4^Div. Paediatric Haematology & Oncology, ^5^Div. of Paediatric Rheumatology, ^6^Div. of Paediatric Pulmonology, ^7^Div. of Paediatric Gastroenterology, ^8^Neonatal Intensive Care Unit, ^9^Paediatric Intensive Care Unit Hauner Children’s Hospital, University Hospital, Ludwig Maximilians University (LMU), Munich, Germany, ^10^Department Pediatric Cardiology and Pediatric Intensive Care, University Hospital, Ludwig Maximilians University (LMU), Munich, Germany

**PARTNER: bioMérieux (France)**

Principal Investigator

François Mallet^1,2, 3^

Research Group

Karen Brengel-Pesce^1,2, 3^

Alexandre Pachot^1^

Marine Mommert^1,2^

^1^Open Innovation & Partnerships (OIP), bioMérieux S.A., Marcy l'Etoile, France

^2^Joint research unit Hospice Civils de Lyon - bioMérieux, Centre Hospitalier Lyon Sud, 165 Chemin du Grand Revoyet, 69310 Pierre-Bénite, France

^3^EA 7426 Pathophysiology of Injury-induced Immunosuppression, University of Lyon1-Hospices Civils de Lyon-bioMérieux, Hôpital Edouard Herriot, 5 Place d’Arsonval, 69437 Lyon Cedex 3, France

**PARTNER: University Medical Centre Ljubljana (Slovenia)**

Principal Investigator

Marko Pokorn^1,2,3^ MD, PhD

Research Group

Mojca Kolnik^1^ MD, Katarina Vincek^1^ MD, Tina Plankar Srovin^1^ MD, PhD, Natalija Bahovec^1^ MD, Petra Prunk^1^ MD, Veronika Osterman^1^ MD, Tanja Avramoska^1^ MD

^1^Department of Infectious Diseases, University Medical Centre Ljubljana, Japljeva 2, SI-1525 Ljubljana, Slovenia

^2^University Childrens' Hospital, University Medical Centre Ljubljana, Ljubljana, Slovenia

^3^Department of Infectious Diseases and Epidemiology, Faculty of Medicine, University of Ljubljana, Slovenia

**PARTNER: Amsterdam, Academic Medical Hospital & Sanquin Research Institute (Netherlands)**

Principal Investigator

Taco Kuijpers ^1,2^

Co-investigators

Ilse Jongerius ^2^

Recruitment team (EUCLIDS, PERFORM)

J.M. van den Berg^1^, D. Schonenberg^1^, A.M. Barendregt^1^, D. Pajkrt^1^, M. van der Kuip^1,3^, A.M. van Furth^1,3^

Students PERFORM

Evelien Sprenkeler ^2^, Judith Zandstra ^2^

Technical support PERFORM

G. van Mierlo ^2^, J. Geissler ^2^

^1^ Amsterdam University Medical Center (Amsterdam UMC), location Academic Medical Center (AMC), Dept of Pediatric Immunology, Rheumatology and Infectious Diseases, University of Amsterdam, Amsterdam, the Netherlands

^2^ Sanquin Research Institute, & Landsteiner Laboratory at the AMC, University of Amsterdam, Amsterdam, the Netherlands.

^3^ Amsterdam University Medical Center (Amsterdam UMC), location Vrije Universiteit Medical Center (VUMC), Dept of Pediatric Infectious Diseases and Immunology, Free University (VU), Amsterdam, the Netherlands (former affiliation)

**References**

1. Love, M.I., W. Huber, and S. Anders, *Moderated estimation of fold change and dispersion for RNA-seq data with DESeq2.* Genome Biology, 2014. **15**(12): p. 550.

2. Kramer, A., et al., *Causal analysis approaches in Ingenuity Pathway Analysis.* Bioinformatics, 2014. **30**(4): p. 523-30.

3. Rigby, R.A. and D.M. Stasinopoulos, *Generalized additive models for location, scale and shape.* Journal of the Royal Statistical Society: Series C (Applied Statistics), 2005. **54**(3): p. 507-554.

4. Roberts, A.G.K., D.R. Catchpoole, and P.J. Kennedy, *Identification of differentially distributed gene expression and distinct sets of cancer-related genes identified by changes in mean and variability.* NAR Genom Bioinform, 2022. **4**(1): p. lqab124.

5. de Jong, T.V., Y.M. Moshkin, and V. Guryev, *Gene expression variability: the other dimension in transcriptome analysis.* Physiol Genomics, 2019. **51**(5): p. 145-158.

6. Newman, A.M., et al., *Determining cell type abundance and expression from bulk tissues with digital cytometry.* Nature Biotechnology, 2019. **37**(7): p. 773-782.
